# The evolution of secondary flow phenomena and their effect on primary shock conditions in shock tubes: Experimentation and numerical model

**DOI:** 10.1371/journal.pone.0227125

**Published:** 2020-01-16

**Authors:** Sudeepto Kahali, Molly Townsend, Melissa Mendez Nguyen, Jeffrey Kim, Eren Alay, Maciej Skotak, Namas Chandra

**Affiliations:** 1 Department of Mechanical Engineering, New Jersey Institute of Technology, Newark, New Jersey, United States of America; 2 Department of Biomedical Engineering, New Jersey Institute of Technology, Newark, New Jersey, United States of America; Coastal Carolina University, UNITED STATES

## Abstract

Compressed gas-driven shock tubes are widely used for laboratory simulation of primary blasts by accurately replicating pressure profiles measured in live-fire explosions. These investigations require sound characterization of the primary blast wave, including the temporal and spatial evolution of the static and dynamic components of the blast wave. The goal of this work is to characterize the propagation of shock waves in and around the exit of a shock tube via analysis of the primary shock flow, including shock wave propagation and decay of the shock front, and secondary flow phenomena. To this end, a nine-inch shock tube and a cylindrical sensing apparatus were used to determine incident and total pressures outside of the shock tube, highlighting the presence of additional flow phenomena. Blast overpressure, impulse, shock wave arrival times, positive phase duration, and shock wave planarity were examined using a finite element model of the system. The shock wave remained planar inside of the shock tube and lost its planarity upon exiting. The peak overpressure and pressure impulse decayed rapidly upon exit from the shock tube, reducing by 92–95%. The primary flow phenomenon, or the planar shock front, is observed within the shock tube, while two distinct flow phenomena are a result of the shock wave exiting the confines of the shock tube. A vortex ring is formed as the shock wave exited the shock tube into the still, ambient air, which induces a large increase in the total pressure impulse. Additionally, a rarefaction wave was formed following shock front expansion, which traveled upstream into the shock tube, reducing the total and incident pressure impulses for approximately half of the simulated region.

## Introduction

For a few centuries, the explosive blast was a threat to soldiers and civilians in armed conflicts. Only in the early days of the 20^th^ century, technological advances in the weaponry and massive deployment of artillery in the trench warfare during World War I resulted in the identification of the neurological and psychological effects of blast waves, described as the shell shock [1]. The mechanisms responsible for shell shock were poorly understood; the term was subsequently banned and replaced with post-concussive syndrome during World War II [2]. It was only a decade after WWII when systematic research to identify mechanisms responsible for blast injuries was initiated. Blast lung injury was the subject of intense experimental study at the Lovelace Foundation in Albuquerque, New Mexico from the 1950s to 1980 [3]. The research group led by Clemedson was among the first to use blast tubes to study the effect of shock waves on biological tissues [4]. Both groups used shock tubes, which had previously been used in other research fields to study detonation, combustion, ionization, supersonic, and transonic flow fields since the early 19^th^ century [5–8]. In the last two decades, shock tubes have become a standard laboratory tool to study, the effect of shock waves on animal models, including rodents, pigs, and ferrets [9, 10]. In a broad survey of recent literature (time span: 2010–2019, S1 Table), trends can be observed in 71 experimental studies using shock tubes [11].

**Fig 1:**
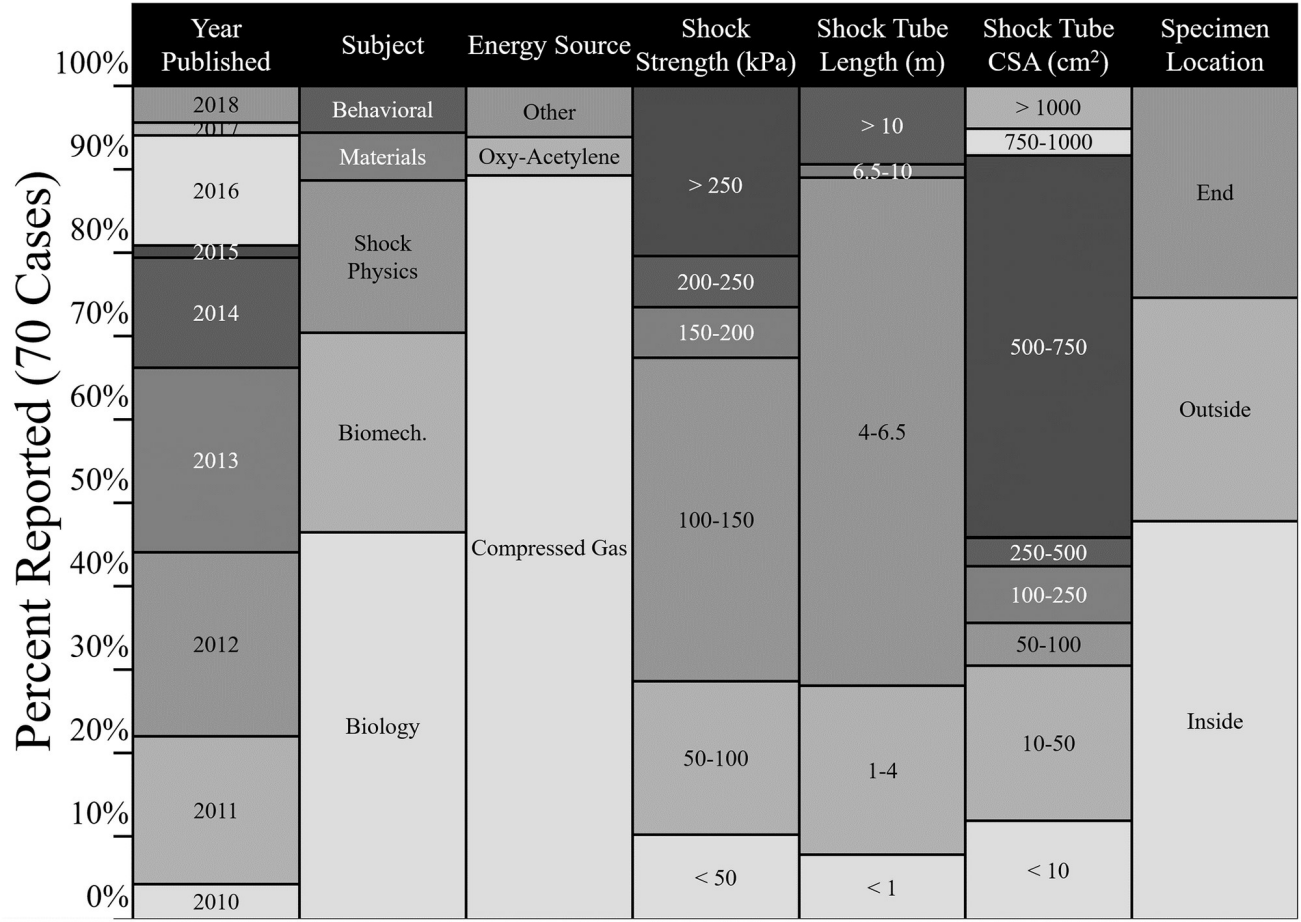
Summary of a recent literature survey of experiments using shock tubes. The survey analyzed 71 recent experiments, published between 2010 and 2018, which were conducted using a shock tube. The experimental subject being investigated, the energy source used to generate the shock wave, the shock strength, shock tube length, and size, and the location of specimens were identified for each study. Biomech.: Biomechanics; CSA: cross-sectional area. See S1 Table for additional details.

The survey, summarized in Fig 1, shows that in the majority of the work surveyed, the purpose of the work was to study injury pathologies in animal models (76%). In more than half of the tests surveyed, the specimens (animal models, human surrogates, or animal surrogates) were tested near the exit or outside of the shock tubes. However, it is well-established that an unconfined shock front will undergo diffraction upon exit from the shock tube [11]. Free expansion of the unconstrained shock front at the exit induces a flow field which is very different from that of the inside; the flow field is affected by blast winds, air entrapment, vorticity, and rarefaction waves [12, 13]. An object placed at different locations both inside, near the exit, or outside of the shock tube is subjected to different types of loadings based on the static and dynamic components of the total pressure of the flow field.

Experimental investigation into the hydrodynamica of shock wave diffraction from an open ended shock tube utilize a variety of optical techniques to visualize the flow field. Some optical techniques include particle image velocimetry [14], holographic interferometry [15], schlieren imaging [16], smoke flow visualization [17]. These techniques capture the density or instantaneous velocities of the flow fields and are primarily used to understand and characterize the nature of exit jets, vortex rings. Increasingly, these optical techniques are coupled with numerical simulations to better describe the observable flow phenomena, enabling additional quantifiable analysis of the flow field [18]. However, despite the wealth of work performed in this field, a majority of the work conducted is conducted in shock tubes which are much smaller [11, 14, 19, 20] or much larger [21] than those conventionally used in biomedical applications. Additionally, much of the work is conducted at Mach numbers which would induce a fatal injury in animal models, precluding their usefulness for the study of mild traumatic brain injury [22–25].

In this work, we have generated shock waves at three shock strengths which have been previously shown to generate mild traumatic brain injuries in animal models [26]. For each of those conditions, we have made sixteen measurements of static and dynamic pressures in separate experiments. Additionally, we have developed a computational model to simulate the flow field produced in the shock tube experimental setup and validated the computational model against experimental measurements. Based on additional simulations, we have identified the temporal and spatial evolution of flow phenomena resulting from the free expansion of an unconstrained shock. It confirms the earlier evidence that experiments conducted outside of the shock tube do not ever reproduce the idealized flow field conditions of primary shock waves [27, 28].

## Methods

### Validation dataset

**Fig 2:**
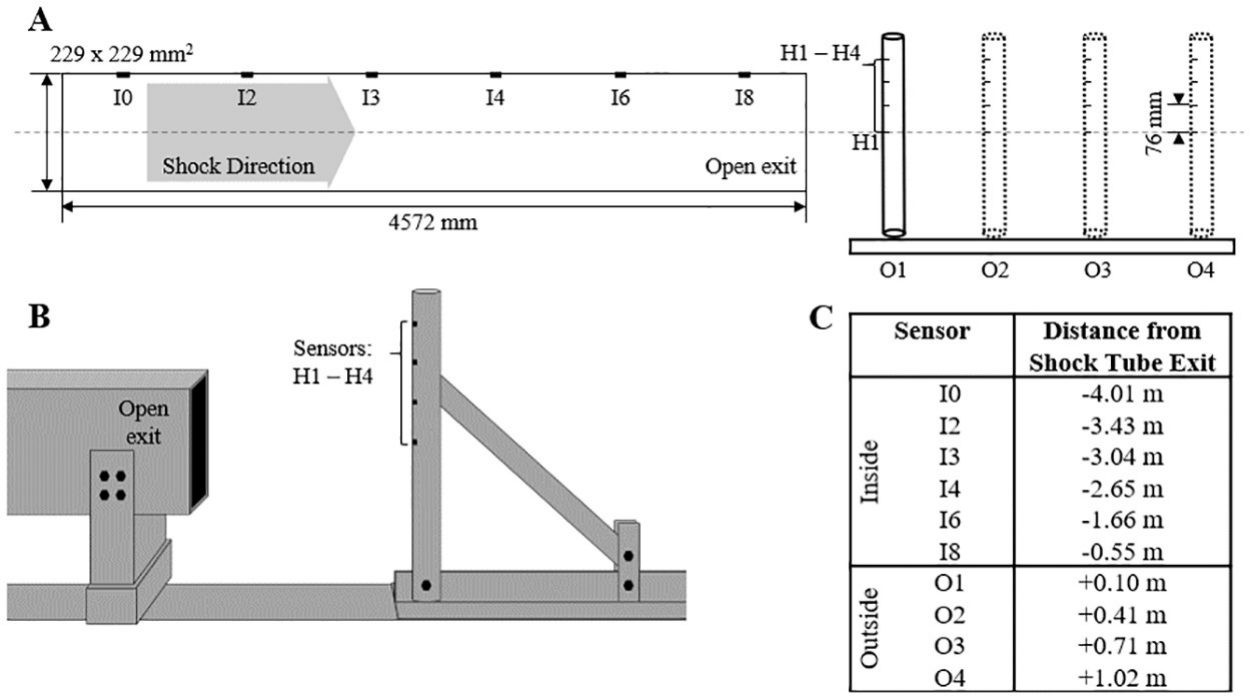
Schematic of the experimental setup used to create the validation dataset. (A) A shock tube with a square cross section was used in the development of the experimental dataset. Incident pressures were measured at six locations within the shock tube (I0, I2-I4, I6, and I8). Total and incident pressures were measured at four longitudinal locations outside of the shock tube (O1-O4) at four vertical heights (H1-H4). (B) A schematic representation of the experimental setup for the O1-O4 tests, with the sensing apparatus in the total pressure orientation. (C) The locations of all sensors with respect to the shock tube exit. Negative values denote upstream distances, into the shock tube, and positive values indicate downstream distances.

The shock tube used in this work has been validated to reproduce free-field explosions within the test section accurately at sensor location I3 in Fig 2 (230 x 230 mm^2^ square cross-section, 6 m in length) [29, 30]. Briefly, compressed helium within a 55 cm length, 10 cm diameter chamber is separated from atmospheric-pressure air by Mylar membranes. When the pressure differential causes the membranes to burst, a shock wave is formed which travels through a 1.5 m transition region to the driven region. Shock strength is dependent upon the pressure ratio between the pressurized chamber of helium and the body of ambient air. In this work, three pressure ratios, 15.8:1, 8.7:1, and 2.5:1, were used to generate a shock wave with overpressures within the test section of the shock tube of 180, 130, and 70 kPa. These overpressures induced shock waves at the test section traveling at Mach 1.2–1.44 and at the shock tube exit at Mach 1.15–1.29. As this work seeks to study the evolution of the shock wave as a function of overpressure, the shocks will be referred to as a high, moderate, and low strength shock, respectively.

Incident pressures were measured within the shock tube at six locations (Fig 2A and 2C) using flush-mounted piezoelectric pressure sensors (Model 134A24, PCB Piezoelectronics). Additional incident pressure measurements and total pressure measurements were taken outside the shock tube, in line with the longitudinal axis of the shock tube as depicted in Fig 2A. The PCB 102B06 pressure sensors were mounted in an aluminum cylinder referred to as the sensing apparatus (61 cm length, 51 mm diameter, 6.4 mm wall thickness), rigidly attached to the shock tube support structure. Four pressure sensors were placed on the upper half of the sensing apparatus at four heights (H1-H4), with the H1 sensor aligned with the longitudinal axis of the shock tube and an inter-sensor spacing of 76 mm. The sensing apparatus was positioned at four locations offset from the exit (open end) of the shock tube, at 4, 16, 28, and 40 in (100, 410, 710, and 1020 mm; O1-O4). Total pressures were measured by orienting the pressure sensors so that the longitudinal axis of the pressure sensor was parallel to the longitudinal axis of the shock tube. Although this technique explicitly measured the reflected pressures on the surface of the sensing apparatus, the reflected pressure is known to decay rapidly to the total pressure due to wave interference from rarefaction waves generated from the incident wave diffracting about the cylinder and, therefore, can be assumed to closely approximate the total pressure [31]. Incident pressures were measured by rotating the sensing apparatus 90 degrees, such that the sensors were perpendicular to the longitudinal axis of the shock tube. Additional incident pressure measurements were taken within the shock tube at six locations along the shock tube (I0, I2-I4, I6, and I8). Experiments were repeated four times (n = 4) at each measurement location (O1-O4), for each measurement type (incident and total), at the three shock strengths (high, moderate, and low).

### Finite element model

**Fig 3:**
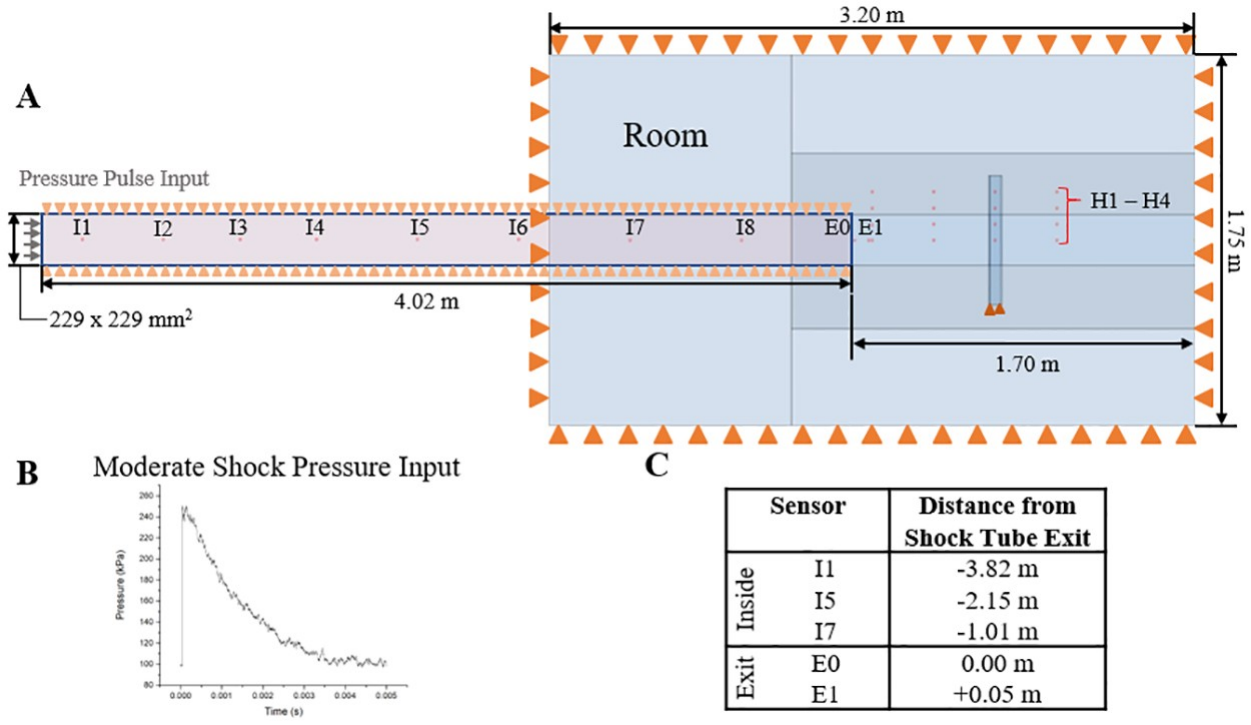
Schematic of the experimental simulation setup. (A) A depiction of the modeling domain, consisting of the sensing apparatus and the shock tube and room region with boundary conditions. Orange triangles indicate boundaries which are constrained. (B) An example of the pressure-time amplitude used to induce the moderate shock wave, which consists of the average pressure measured at sensor location I0 from Fig 1 (n = 4). (C) A table of additional sensor locations which were included in the numerical simulations. The sensor locations are reported with respect to the shock tube exit. Negative values denote upstream distances, into the shock tube, and positive values denote downstream distances.

The finite element method was used in this work to simulate the generation and propagation of the shock wave. A three-dimensional Eulerian model of the experimental setup was created and consisted of two continuous domains, a 4 m segment of the shock tube and a room region surrounding the exit of the shock tube (3.2 x 1.75 x 1.75 mm^3^). The length of the shock tube was selected to simulate the portion of the shock tube located downstream from the first sensor location (I0). A sensitivity analysis was conducted to identify the dimensions of a room region which optimizes computational time and solution quality, where the shock wave would not be affected by any reflections from the boundary of the room (see S1 Fig). The Eulerian domain was discretized using a biased mesh with a minimum element edge length of 8 mm at all regions of interest (see S2 Fig). To validate the simulation, a Lagrangian model of the sensing apparatus was created to match the experimental setup. Fig 3A shows the shock tube, room region, and the sensing apparatus with the testing locations used for model validation. The Lagrangian domain offered a converged solution at an ideal mesh density of 6 mm (see S3 Fig). This resulted in a system of approximately 3,600,000 Eulerian and 3,500 Lagrangian isothermal, reduced integration, linear, hexahedral elements with hourglass control. The Eulerian domain was filled with 300 K air at a density of 1.225 kg/m^3^. The sensing apparatus was modeled as aluminum, approximated to be linear, elastic, and isotropic with a density, elastic modulus, and Poisson’s ratio of 2700 kg/m^3^, 70 GPa, and 0.33, respectively.

A typical experimental pressure-time profile collected from the sensor I0 (Fig 3B) was used to model the shock wave. Although experiments were shown to be very reliable (see S4 Fig), the pressure-time pulses of a minimum of four exposures were averaged for each shock strength to minimize the effect of any measurement artifacts. The edges of the room and shock tube were constrained against displacement normal to the edge (Fig 3A). The open exit of the shock tube was unconstrained, allowing for the unimpeded flow of the shock wave from the shock tube into the room region.

Simulations to derive the incident pressures were conducted without the sensing apparatus model in a purely Eulerian simulation. Total pressure simulations were conducted with the sensing apparatus. In the total pressure simulations, all nodes located at the bottom of the sensing apparatus were constrained in all translational and rotational degrees of freedom. The contact between the Lagrangian and Eulerian domains is based on an enhanced boundary method. Here, the void mesh of the Eulerian domain is occupied by the Lagrangian structure. The general contact algorithm automatically tracks the interface between the domains, compensating for mesh size discrepancies to prevent the entry of Eulerian material through the Lagrangian surface. Contact constraints are enforced through the penalty method with a finite sliding contact formulation. The total pressure simulations use frictionless tangential sliding with hard contact.

All simulations were performed using Abaqus/Explicit 6.13–4 (Simulia, Dessault Systemes) in 16 threads of an 3.00 GHz Intel i7-5960X with 64GB of physical memory.

### Methodology

Overall thirty-nine simulations were conducted, three incident pressure simulations, one for each shock strengths (low, moderate, and high), and thirty-six total pressure simulations at three shock strengths for twelve locations. Additional locations were identified to better map the shock tube system, including seven locations inside the shock tube (I1-I8), two locations near the exit of the shock tube (E0 and E1), and four locations outside the shock tube (O1-O4). Exact locations of the additional measurements are included in Fig 3C.

First, validation was conducted in which the shock wave pressure-time profiles were compared to the experimental measurements. The peak overpressure, impulse, duration, and general form of the total and incident pressure-time profiles were compared to ensure validation of the simulation. Upon validation, the simulation results were analyzed to identify potential flow phenomena.

## Results and discussion

In the discussion below, the evolution of a shock wave at the exit of a shock tube is presented, including the discussion of how the shock wave decays in strength and induces several flow phenomena. First, the observations from the experimental measurements will be presented and the numerical simulations will be validated. Then, the numerical model, supported by experimental measurements when possible, will be used to discuss several metrics used to define the characteristics of the shock wave. First, the shock is observed to be planar within the shock tube and non-planar outside of the shock tube. The shock tube constrains the shock wave, maintaining the planarity of the wave until the shock front experiences rapid expansion into the ambient air within the room. Next, the peak pressure decays as the shock wave propagates, decreasing slowly while inside the shock tube and decreasing rapidly upon exiting the shock tube. Finally, two flow phenomena were generated from the rapid expansion of the shock front at the shock tube exit: a vortex ring and a rarefaction wave. The vortex ring propagates behind the shock wave at a slower velocity that is dependent on the shock strength. The rarefaction wave reflects into the shock tube, decreasing the positive phase duration and impulse. These metrics of the shock wave highlight the regions of the shock tube experimental setup which experience an ideal shock wave and those which interact with a vortex ring and a rarefaction wave.

### Experimentally observed incident and total pressures

**Fig 4:**
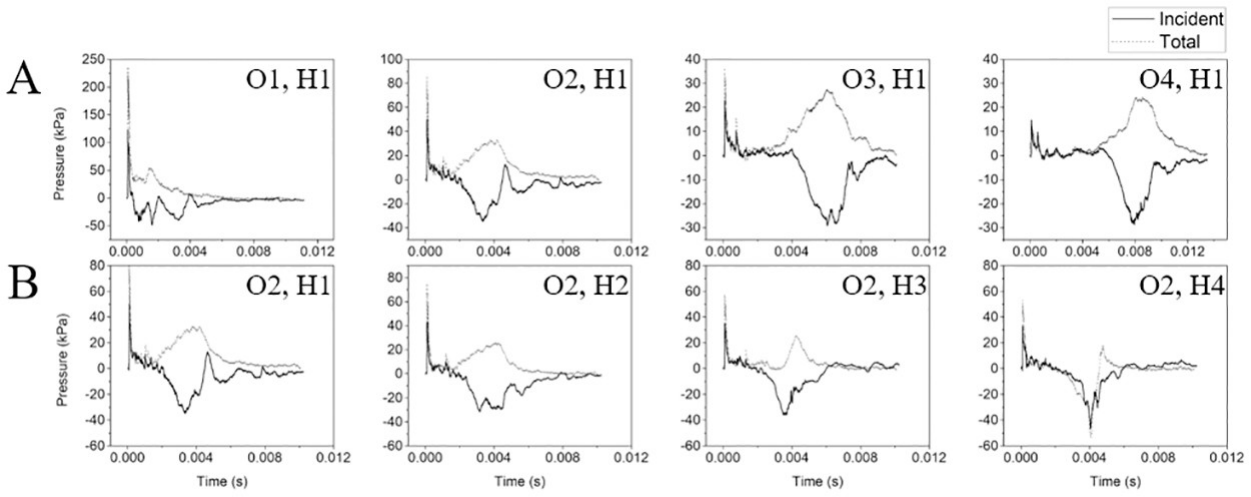
Measurement location along the longitudinal axis (O1-O4) and the vertical axis (H1-H4) changes the characteristics of the pressure measurements. (A) The experimentally measured total (grey) and incident (black) pressures at the four measurement locations O1-O4 at sensor location H1 highlight a reduction in the peak overpressure and a delay in the arrival of the secondary flow phenomena as the distance from the shock tube exit increases. (B) Likewise, when comparing the pressure profiles vertically (H1-H4) at a single longitudinal location, O2, shows a change in the nature of the secondary flow phenomena.

Experimental measurements of the shock overpressure outside the shock tube exit identified two distinct flow phenomena (Fig 4). First, the shock front arrives, characteristically observed as a rapid increase in overpressure and an exponential decay to baseline in which the reflected pressure is proportionally higher than the incident pressure reading. The reflected pressure rapidly decays to the total pressure, which remains proportionally higher than the incident pressure reading. The difference between shock front arrival time can be used to confirm the shock velocity, which is confirmed to be higher in higher shock intensities and gradually slows as it propagates in the ambient air outside the shock tube. The peak overpressure of the shock front decreases as the distance from the shock tube exit increases. Additionally, the peak overpressure decreases with increased distance from the midline, i.e., normal to the direction of shock wave propagation. This trend is less pronounced as the distance from the shock tube exit increases.

The second distinct flow phenomenon arrives after the shock front and is characterized by a slow increase in the total pressure and decrease in the incident pressure, resulting in an incident underpressure, or pressure less than atmospheric pressure. At a distance closest to the shock tube exit, O1, the flow artifact occurs shortly after the shock passes. This artifact is observed to travel at a slower velocity than the shock front, moving at a subsonic velocity with a magnitude proportional to the shock strength. As these rarefaction waves travel at the speed of sound within the fluid behind the shock front, a subsonic velocity is observed. This flow phenomenon was observed at sensor locations H1-H3 and the highest sensor measurement location, H4, showed an underpressure in both the total and incident pressures.

**Fig 5:**
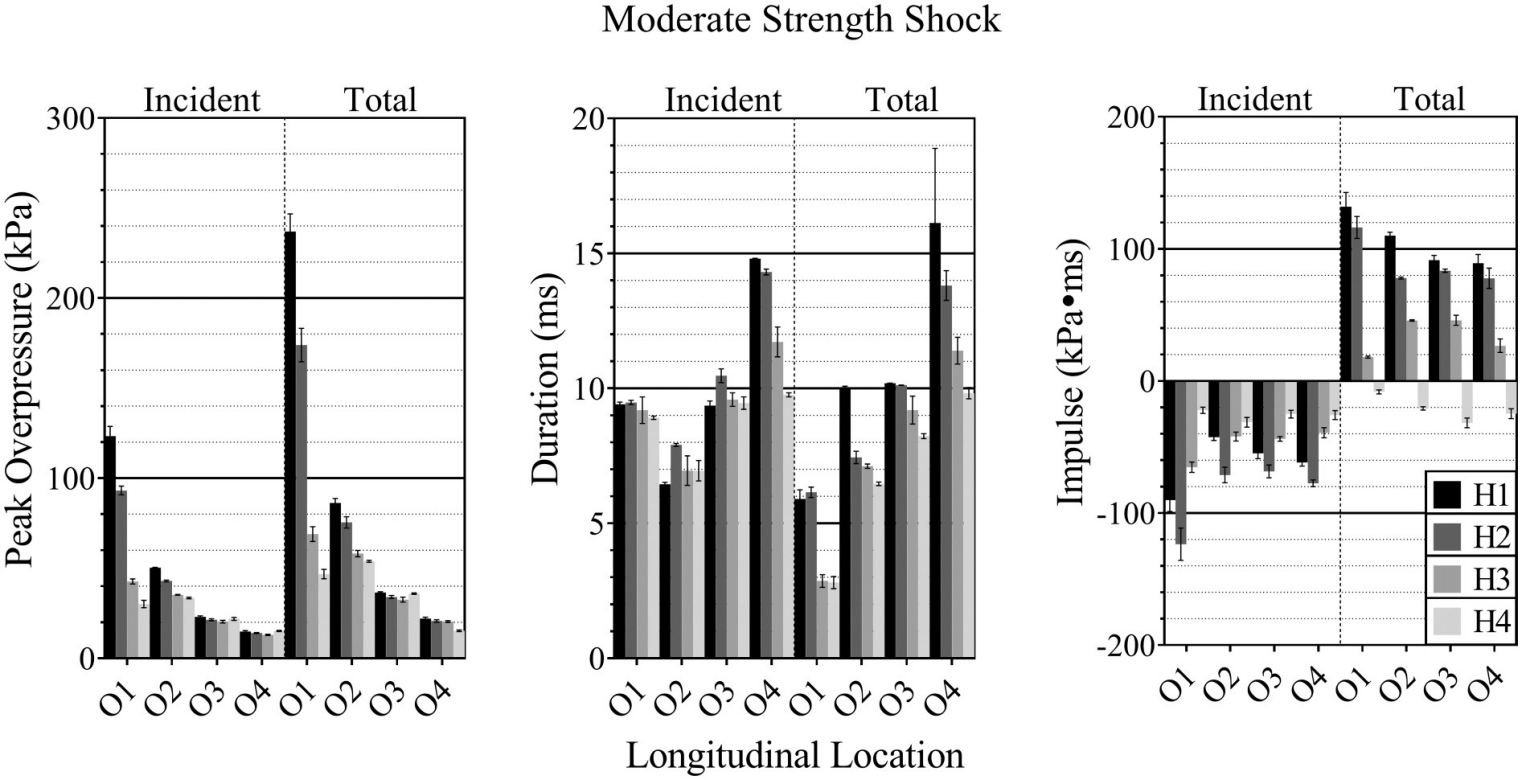
Pressure profile characteristics of the incident and total pressures. The peak overpressure (left), signal duration (middle), and impulse (right) of the experimentally measured incident, and total pressure pulses for the moderate-intensity shock wave showed several trends.

A few general trends can be observed in Fig 5 by comparing the peak overpressure, positive phase durations, and the impulse at the four vertical locations (H1-H4) at the four longitudinal sensing apparatus measurement locations (O1-O4). Trends are conserved between the three shock strengths examined, differing only in magnitude (Fig 5). Peak overpressure, duration, and the magnitude of the impulse increased with shock strength. Over the duration of the pressure signal, underpressures resulted in negative impulse values in all sensor locations in the incident waveform and in the O4 total pressure measurement. Total pressure measurements exhibited higher peak overpressures and impulses in all cases. As longitudinal distance from the shock tube exit increased (O1-O4), peak overpressure and impulse decreased. There exhibited no significant change in incident pressure duration and the total pressure duration decreased with increasing longitudinal distance. As the vertical distance increased from the longitudinal axis (H1-H4), the peak overpressure decreased, with the magnitude of the reduction decreasing with longitudinal distance. Significance was not observed between the H1 and H2 sensors in the incident pressure measurement at O4. The duration of the total pressure signal increased in the off-axis locations.

### Validation of computational model

**Fig 6:**
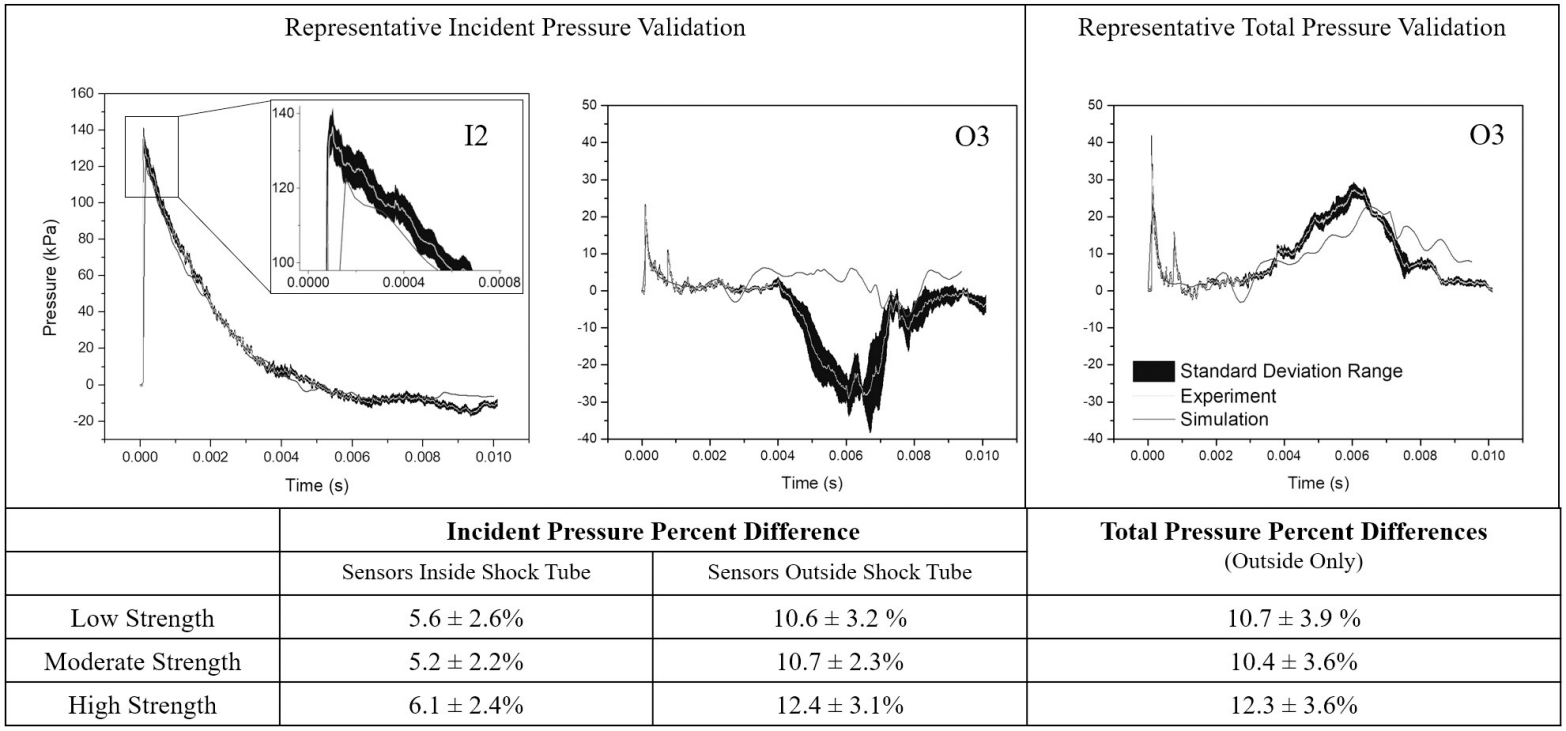
Simulation predictions compared to experimental results for the high-strength shock. The simulated pressures (grey) showed good validation with the average experimentally measured pressures (black with grey, mean, with standard deviation) for the incident (left and middle) and total (right) pressure measurements for the high strength shock. The point-by-point percent error for the shock front was under 12.5%. Average errors for interior locations (I2-I4, I6, and I8) showed the best validation.

The simulations were validated against the experimental datasets through a comparison of the pressure-time waveforms, the peak overpressures, impulses, rise-times, and durations. The simulations, on average, underestimated the peak overpressure and overestimated the rise time, which is attributed to a sampling rate and an element density which were not adequate to resolve the shock front location. The simulations were sampled at a frequency of 26 kHz. Experimentally, sampling frequencies of a similar range have been shown to be sufficient to resolve the peak overpressure, yet overestimate the rise time of the signal [32]. However, low sampling frequency coupled with spatial discretization reduces the ability to capture the shock front location. For the shock strengths simulated, the velocity of the shock front is 345–518 m/s (low to high strength) which would enable the shock front to traverse an 8 mm element in the range of 15.5–20.4 μs, respectively. To accurately resolve the shock front at that spatial density, a higher sampling frequency, coupled with adaptive mesh refinement techniques, would be required. However, as the accurate resolution of the rise time was not the focus of this work, additional techniques were not implemented to improve the simulation of the rise time and the idealized peak overpressure. Despite the underprediction of the peak pressure, average point-by-point percent error between the experimental and simulated pressures were under 6% for all incident pressures within the shock tube and under 12.5% for all incident and total pressure measurements taken outside of the shock tube (Fig 6). On average, the best match was observed at locations closer to the input location, I0.

Simulation results matched the impulse and duration of the shock front, but incident pressure simulations did not exhibit a negative impulse, as seen in the experimental measurements (Fig 5). Several methods of simulating the incident and total pressures were compared, indicating that the decrease in pressure was likely due to the interactions between the secondary flow phenomenon and the cylindrical sensing apparatus (S5 Fig). In general, a comparison of the pressure-time waveforms revealed an excellent match, meaning that the simulation can be considered validated with conservative blast overpressure estimates.

### Peak overpressure

**Fig 7:**
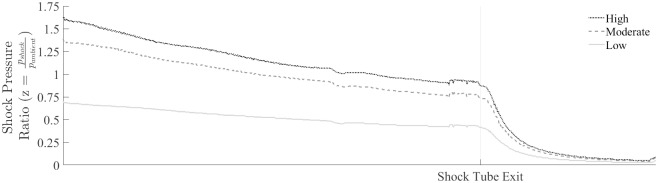
The shock pressure ratio along the longitudinal axis. The shock pressure ratio, *z*, defined as the ratio of the shock front, *p*_*shock*_, to the ambient pressure, *p*_*ambient*_, decays gradually along the shock tube and declines rapidly after expansion begins at the shock tube exit. The rate of decay within the shock tube and the following expansion was found to depend on the shock strength.

The shock pressure ratio, defined as the ratio of the pressure within the shock front and the ambient pressure, decreased slowly as it propagated through the shock tube and rapidly upon sudden expansion into the room region (Fig 7). The rate of decay varied with shock strength, with the high strength shock pressure ratio decaying the most rapidly. The shock pressure ratio decreased within the shock tube at an average rate of 0.09, 0.19, and 0.22 for the low, moderate, and high strength shocks, respectively. The peak pressure at the shock tube exit was 30.99%, 33.33% and 34.15% of the input peak overpressure for the low, moderate, and high strength shock. These results are in line with previous findings from our group, which states that the peak pressure decays as the shock wave propagates down the shock tube [29, 32]. Energy loss occurs due to the expansion of the high-pressure shock front as it interacts with low-pressure upstream air, increasing the shock duration while maintaining a comparable impulse [29].

Shock wave expansion at the shock tube exit decreased the shock pressure ratio rapidly, with the average rate of decay increasing to 0.55, 1.00, and 1.19 and an average peak rate of decay of 2.65, 4.96, and 6.03 for the low, moderate, and high strength shocks, respectively. Outside of the shock tube, the pressure decayed rapidly, reducing 92.96%, 94.33%, and 94.51% for the low, moderate, and high strength shocks, respectively. This rapid decay in overpressure is consistent with an increased area of the shock front as the shock front experiences expansion into the ambient air [33]. A relationship exists between the shock strength and the area of the shock front using the function derived by Chisnell (*f(z)*) which, when multiplied by the area of the shock front (*A*), remains constant [34]. As the area of the shock front increases, the Chisnell function decreases, accordingly, and is given for a shock in diatomic air by [34]

$$f(z)=\frac{z^{\frac{5}{7}}(z-1)}{\sqrt{z+\frac{1}{6}}}{\left[\frac{1+{\left(1+\frac{6}{z}\right)}^{-1/2}}{1-{\left(1+\frac{6}{z}\right)}^{-1/2}}\right]}^{\sqrt{7}/2}\times [\frac{{\left(1+\frac{6}{z}\right)}^{-1/2}-7^{-1/2}}{{\left(1+\frac{6}{z}\right)}^{-1/2}+7^{-1/2}}]\times e^{\left(\sqrt{5}{\text{tan}}^{-1}\left(5\sqrt{\frac{7z}{5(z+6)}}\right)\right)}.$$ (1)

This function is derived from the Rankine-Hugoniot equations and has been proven to accurately model symmetrical, spherical and cylindrical shocks experiencing rapid, unconfined expansion between two square or circular chambers of similar or very different cross-sectional areas[34, 35]. When used in conjunction with the area of the shock front given for a spherical shock by

$$A=2\pi X^{2}$$ (2)

where *X* is the longitudinal distance from the exit of the shock tube after which a critical shock is formed. It can be calculated from the shock tube diameter (*d*) and the angle of propagation (*α*) by

$$X=\frac{d}{2}\text{cot}\;\propto .$$ (3)

Using a relationship between the angle of propagation and the Mach number, given by

$${\text{tan}}^{2}\propto =\frac{\left(M^{2}-1)(M^{2}+5\right)}{6M^{4}},$$ (4)

a relationship describing the loss in shock pressure ratio due to expansion can be derived. Using this theory for the conditions studied here, the rapid decay of the shock pressure ratio is explained by the increase in the area of the shock front. As the shock front area increases and continues to expand, the shock pressure ratio exponentially decays.

### Shock planarity

**Fig 8:**
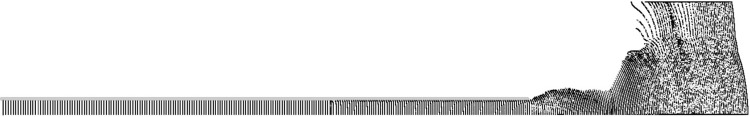
The planarity of the shock front is largely lost at the shock tube exit. The edge of the shock front at each time step (Δt = 38.5 μs) from t = 0–13.5 ms in the moderate strength shock in a mid-wall cut. As the shock exits the shock tube, denoted by a grey bar, the shock front loses its planar nature and expands, becoming non-planar.

As the simulated shock propagated through the shock tube, it remained planar. At the shock tube exit, the shock front experienced sudden, unconstrained expansion into the still ambient air. At this point, the simulation shows the shock front became less planar and expanded. As the shock front propagated, it eventually regained some planarity (Fig 8).

**Fig 9:**
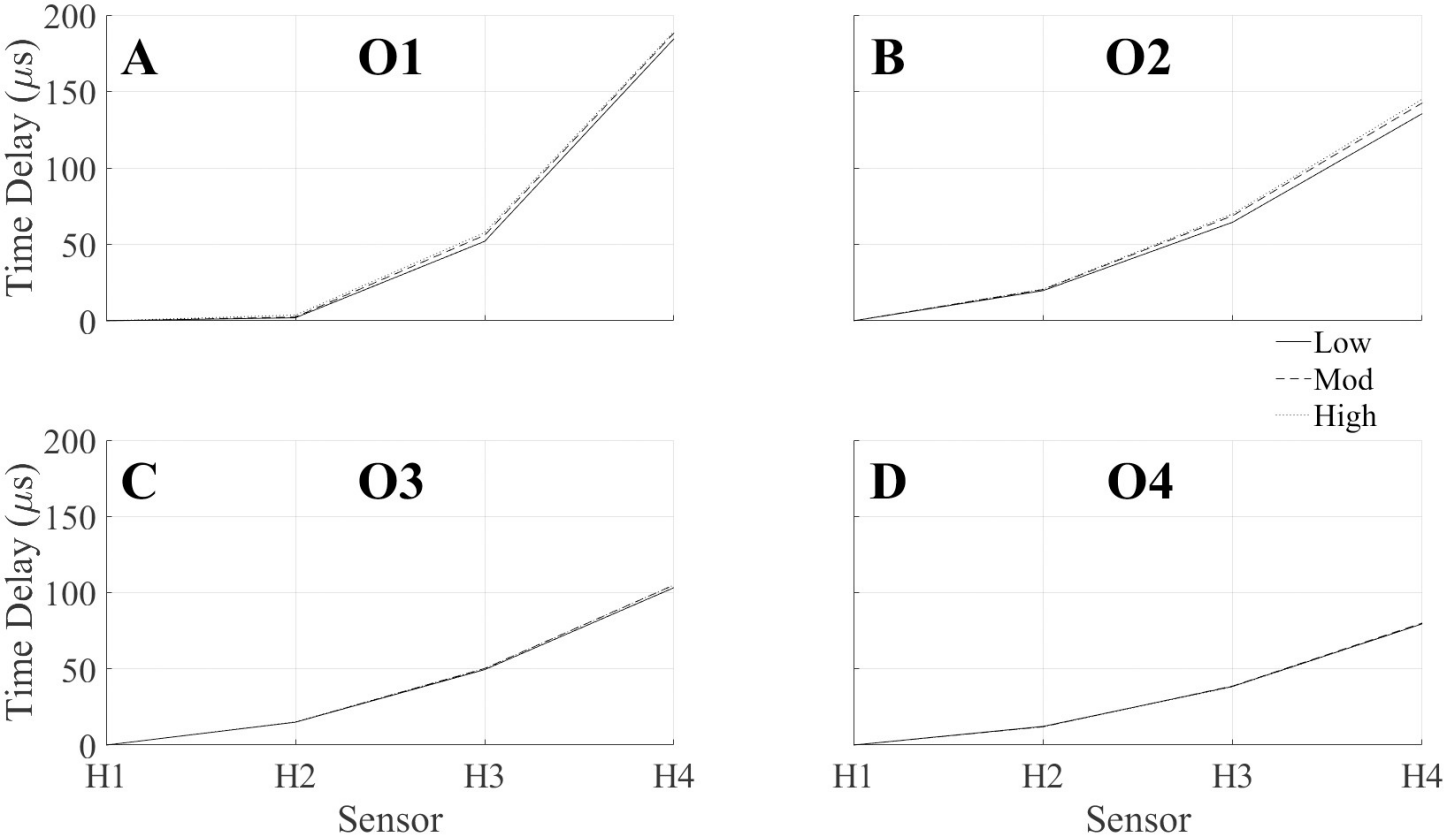
Experimentally observed shock curvature. The arrival times of the shock wave normalized with the shock arrival at the H1 sensor showed a curvature of the shock front at the measurement locations (A) O1, (B) O2, (C) O3, and (D) O4.

This simulated phenomenon was confirmed experimentally by examining the shock front arrival times at each sensor. At all longitudinal measurement locations (O1-O4), the shock wave arrived at the sensor aligned with the longitudinal axis first (H1). At the measurement location closest to the shock tube exit, the center of the shock front remains mostly planar. When comparing the elapsed time between the shock wave arrival, little difference was observed between the two sensors closest to the longitudinal axis, H1 and H2. The arrival of the shock front at H2 was delayed an average of 2.75±0.98 μs. The shock wave curvature is apparent in the much higher arrival time delays at the sensors located farther from the midline, with H3 and H4 being delayed by 55.25±1.91 μs and 187.33±2.12 μs, respectively. This demonstrates that the shock wave is only beginning to lose planarity at this location. The curvature of the shock wave is more apparent at the O2-O4 longitudinal measurement locations, where the shock curvature is more even (Fig 9). The curvature is highest at O2, then starts decreasing to O4. The shock front arrival time delay was mostly independent of the shock strength. A slight trend was observed where a high-strength shock exhibited a slightly longer delay. This trend was most apparent at the O1 location, and the strength of the trend decreased with the distance from the shock tube exit. The shock front travels at a faster speed in the higher strength shock and, therefore, the shock front will be less planar than a low strength shock, despite there being little to no difference in the shock front arrival time delay.

These observations highlight that the shock front was behaving in line with previous experimental results of sudden shock wave expansion, further validated by the theory. Using the relationship in Eq 4, the Mach number of the shock wave as it exits the shock tube can be used to predict the angle of propagation of the critical shock, predicting the point at which planarity of the shock front decays. The difference in the experimentally measured arrival times of the shock front between the I8 and O1 and the distance between those measurement locations were used to calculate the shock velocity (*v*) and the Mach number, using the relationship

$$M=\frac{v}{c},$$ (5)

where the speed of sound, c, was assumed to be 343 m/s. At the measurement location O1, the angles of propagation for the shock strengths investigated in this study are 69.5, 62.9, and 61.8 mm for the low, moderate, and high strength shocks, respectively. This indicates that the limits of the planar area fall between the H1 and H2 sensors (76.2 mm) for all three shock strengths at the O1 location, as demonstrated by a lack of curvature seen in Fig 9A. The point of contact, or the point in which the shock loses the original planar shock front, occurs between 222–374 mm for the shock strengths examined in this study. Therefore, by the O2 longitudinal measurement location, the shock front no longer retains any planarity and decays completely, referred to as a “critical shock”. As the shock expands into the ambient air, the shock undergoes diffraction and expands freely around the sharp edge of the shock tube exit. This shock front which undergoes diffraction interacts with the planar shock front, causing the planar front to decay into a critical shock. This interaction creates an expansion wave which propagates longitudinally upstream into the shock tube [33]. The expansion wave, or rarefaction wave, will be described in detail later.

Following the complete decay of the planar shock front into a critical shock, the critical shock front was observed in the simulations to propagate at a much lower strength and, eventually, become more planar in nature. This is theorized to be due to the initial uneven expansion of the wave into a critical shock and due to interactions of the shock front with the boundaries of the room region. As the cross-section of the shock tube investigated here is not of a circular diameter, the planar region will decay in a non-axisymmetric manner. The narrower cross section at the mid-wall of the shock tube will have a smaller planar area than the widest cross-section, at a diagonal cut. The non-axisymmetric decay will cause the behavior of the critical shock to deviate from the ideal behavior of a spherical shock. Additionally, as the critical shock continues to expand, the increase in the shock area will eventually interact with the boundaries of the domain. At the domain walls, the interaction of the critical shock with the wall creates an oblique reflected shock, which interacts with the critical shock front [36]. The oblique reflected shock increases the strength of the critical shock, which decreases the apparent curvature of the shock front at the edges of the domain. As the domain of the room region in the computational model was not designed to completely replicate the room in which the experiment was conducted, it is anticipated that the curvature of the shock predicted in the simulation would differ from experimental conditions. However, as the region of interest tested by the sensing apparatus was small in comparison with the room region, it is hypothesized that any differences would only be apparent at the longitudinal measurement locations farthest from the shock tube exit.

### Vortex ring

**Fig 10:**
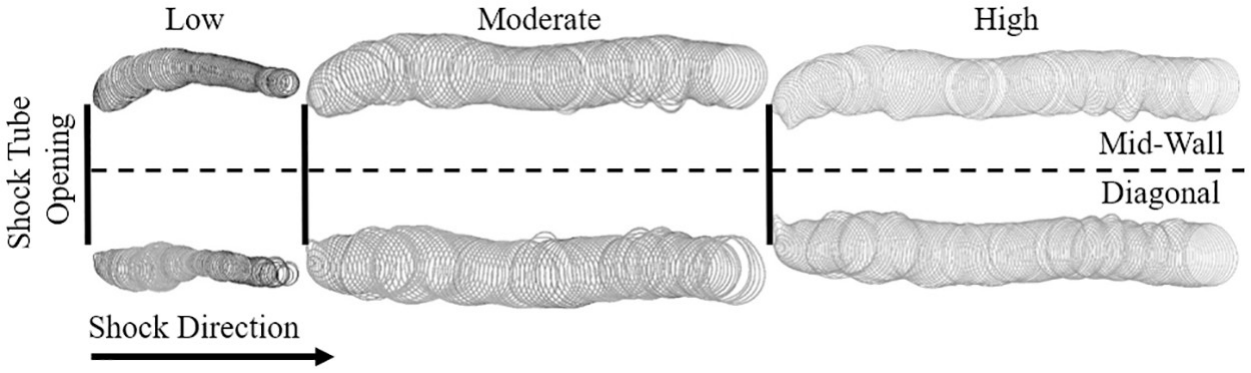
The location of the vortex ring with respect to time. Contour plots of the pressure for a cut through the middle of the shock tube wall (upper) and along the diagonal of the square (lower) display the vortex ring location with time following the expansion of the (left) low, (middle) moderate, and (right) high strength shocks.

Following the shock front, the secondary flow phenomenon observed in the experimental results was identified as a vortex ring. A vortex ring is formed as the fast-moving volume of compressed air moves into stationary air. The energy of the shock front is locally reduced due to the interaction of the shocked and unshocked air, reducing the velocity of the shocked air. The slowed air then moves around the mass of compressed air, then rejoins the fast-moving air, forming a toroidal ring. The simulation showed the rotating ring forming at the exit of the shock tube and following the shock front at a slower velocity. The velocity of the vortex ring was dependent on the shock strength, where the highest strength shock produced a vortex ring which propagates at 90.6 m/s; and with decreasing shock strength, the velocity decreased to 79.8 and 40.3 m/s for the moderate and low strength shocks, respectively. Observed vortex ring propagation velocity aligns well with the approximation where the vortex ring propagation velocity is approximately half of the piston velocity, with theoretical velocities of 83.3, 73.2, and 44.9 m/s for the high, moderate, and low strength shocks respectively [37]. The path of the vortex ring also varied with shock strength (Fig 10). Following the formation of the ring, the vortex ring follows the shock front and the diameter of the vortex ring core at the mid-wall increases to match the diameter of the vortex ring core at the corners. The ring over-expands and comes back to reach a more stable diameter. The diameter of the ring itself increases as it forms and reaches a relatively stable size. The lower severity shock exhibited a reduction in ring size as it propagates, indicating that the ring may have begun to dissipate.

**Fig 11:**
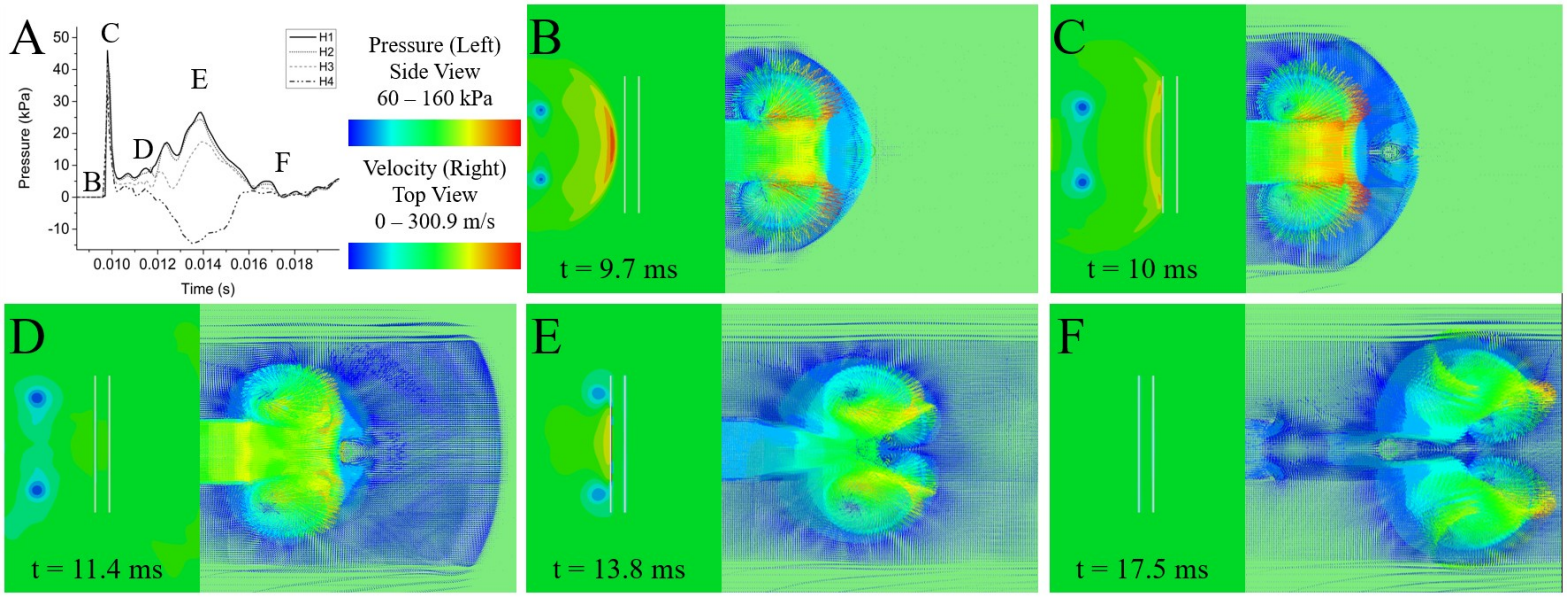
Pressure contours and velocity fields depicting how the vortex ring interacts with the cylindrical sensing apparatus. The interaction of the vortex ring at the O3 location under a moderate-intensity shock, visualized in (left) a side view of the pressure field, shown in MPa, and (right) a top-view of the velocity vectors, shown in m/s for (B) shock front arrival, (C) peak pressure, (D) vortex ring arrival, (E) peak vortex ring interaction, and (F) complete passage of the vortex ring.

At the time of arrival of the vortex ring, an increase in total pressure was observed experimentally and confirmed in the simulation results. A jet of air behind the shock front exists within the core of the vortex ring. This jet of air arrives with the vortex ring, and the air particles within the jet of air are stopped by an object in the path, resulting in an increase in total pressure. The structure of the vortex ring means that the pressure profile varied for each vertical measurement (Fig 11). Along the longitudinal axis, at vertical location H1, the center of the ring accelerated air, which caused the largest total pressure impulse. The impulse decreases with increasing vertical distance, as the sensing location approaches the center of the vortex ring. The vortex ring itself has an underpressure region within the vortex core, where the pressure is falling below atmospheric pressure [38]. The H4 sensor location is closest to the vortex core and characteristically drops in pressure as the vortex ring interacts with the testing apparatus.

### Rarefaction wave

**Fig 12:**
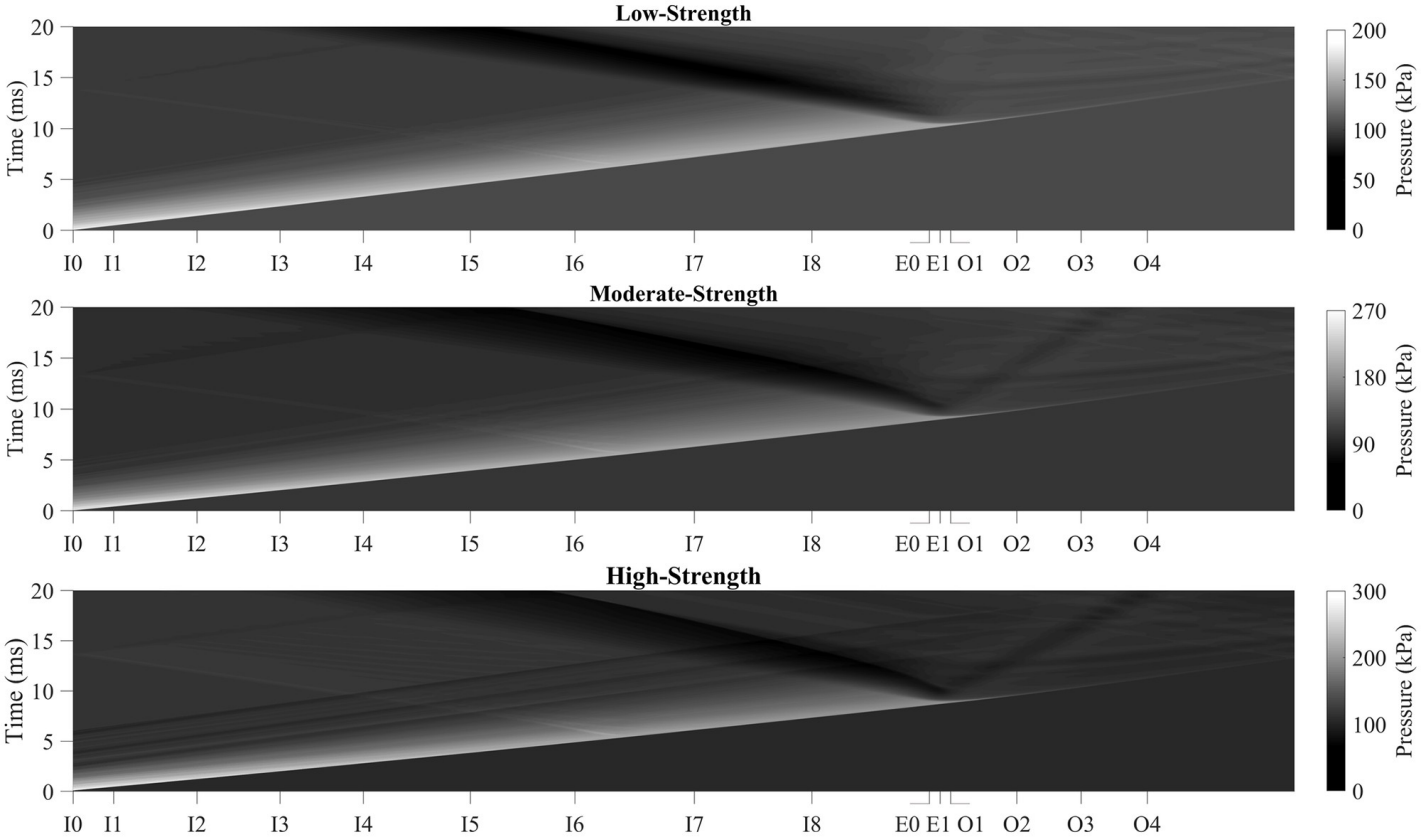
Pressure-time contour plots along the longitudinal axis. A surface map of the pressure for each node along the longitudinal axis plotted as longitudinal location vs. time, shows a rarefaction wave for the (top) low strength, (middle) moderate-strength, and (bottom) high-strength shock. The posteriorly traveling dark fan shows the region of low pressure, which is characteristic of the rarefaction wave.

Another flow artifact was observed following the exit of the shock wave from the confines of the shock tube. A rarefaction wave radiated upstream back into the shock tube, causing as a decrease in blast overpressure. The pressure along the longitudinal axis of the shock tube was mapped with time to capture the nature of this flow phenomenon (Fig 12). After the time point in which the planar shock front exits the shock tube, a fan can be observed which radiates upstream, into the shock tube. The speed of this rarefaction wave and the affected area changes with shock strength, with the rarefaction wave in the low strength shock exhibiting the largest area of influence, with a notable depression observed as deep as sensor I4. The rapid expansion of the shock front at the shock tube exit causes a density gradient to form, which initiates the rarefaction wave. Previously, we have shown that a reflection generated from a reflector plate at an appropriate offset can largely nullify the impact of the rarefaction wave on the incident waveform within the shock tube [29]. A normal reflection of the shock on a perpendicular endplate causes a reflected compressive wave that greatly reduces the effect of the tensile rarefaction wave. However, without an endplate, the tensile wave travels unimpeded and can affect the nature of the pressure waveform.

## Conclusions

The presented work highlights the spatial and temporal evolution of flow phenomena in the shock tube experimental setup relevant to the field of blast-induced traumatic brain injury. Corroberated with pressure measurements and numerical simulations, the nature of these flow phenomena are confirmed and discussed in the range of shock strengths and shock tube dimensions commonly used to generate field-relevant shock exposures to study traumatic brain injury in animal models. In summary, while the shock front is constrained within the shock tube, the shock exhibits strong similarities to a primary blast in the free field. The sudden expansion of the shock front into the free ambient air induces two flow phenomena which initiate changes to the flow system. The sudden expansion causes a vortex ring formation, which develops and moves sub-sonically along the longitudinal axis, following the shock front. The vortex ring and high dynamic pressures are observed as the vorticity forms around the accelerated air within its core. Additionally, a rarefaction wave develops which propagates upstream into the shock tube, which decreases the overpressure, reducing the impulse and duration of the waveform. The shock front expands non-uniformly into the ambient air. This expanded critical shock continues to dissipate energy through expansion and experiences a reduction in peak overpressure and duration. These observations were simulated using a numerical model, validated extensively against experimental pressure measurements. This work strives to better inform the biomedical field of study by identifying the nature and extent of these flow phenomena in the common testing regime.

The limitations of this work are primarily associated with the fidelity of the numerical modeling domain. The room region outside of the shock tube exit was unobstructed by any other objects. This created an idealized flow field which does not reflect realistic experimental conditions, which would introduce reflections which cause the experiment to deviate from numerical predictions. Additionally, only a limited room domain was considered. This was shown to have a potential influence on the evolution of the critical shock, altering how planar the shock front would appear. This simplification enabled for more efficient simulations, but potentially reduced the simulation fidelity of later time points. And finally, the influence of shock tube size and cross-sectional shape were only postulated in this work. This assumption is supported by the findings of other researchers but is not confirmed through a parametric study. Despite these limitations, we are confident in the reported trends.

These observed phenomena are essential to consider in the planning of biomedical shock tube experiments. If the experimental goal is to capture a primary shock waveform, it is recommended that the experimentalist test in locations which are not affected by the passing of the vortex ring or which experience the expansion rarefaction wave. Failure to do so would result in an alteration of the ideal primary shock characteristics. Similarly, if the goal of the experiment is to examine the interaction of an object with a vortex ring, the experimentalist is encouraged to examine the production and evolution of the vortex ring at the shock strengths investigated. The propagation speed of the vortex ring is dependent on the shock strength, and the size of the vortex ring will be dependent on the size and shape of the shock tube. Within the vortex ring, an area with pseudo-blast winds is observed, but the diameter of the vortex ring, if formed, should be compared to the experimental area to ensure that the specimen is not inadvertently experiencing pressure reductions from the vortex ring. Within the shock tube, close to the shock tube opening, regions of decreased incident pressure impulses and durations can be isolated by targeting the area of influence of the rarefaction wave. Therefore, it is recommended that experimentalists gather incident and total pressures at the experimental testing location to capture the nature of the desired flow field.

## Supporting information

S1 Fig Modeling of the room region of the shock tube. Various sizes of the room region the shock tube were modeled. The pressures at the experimental measurement locations O1-O4 were compared. Three room configurations were modeled, (A) the whole room region, (B) a partial section of the room, and (C) a room region only around the exit of the shock tube. The partial room model was selected to minimize computational errors and simulation time.
(TIF)

S2 Fig Convergence study of the shock tube with a 130 kPa shock wave. (A) The pressure at the testing location within the shock tube was plotted against the number of simulated elements. (B) The internal energy in the shock tube with respect to the number of simulated elements. (C) Table comparing the percent change between mesh seed lengths. Convergence was observed by a mesh seed length of 8 mm.
(TIF)

S3 Fig Convergence study of the Lagrangian sensing apparatus exposed to a 130 kPa shock wave. (A) The pressure at the midline of the sensing apparatus, corresponding with the longitudinal axis of the shock tube, was plotted against the number of Lagrangian elements. (B) The percent change between the pressure prediction for varying mesh seed lengths shows convergence at 6 mm.
(TIF)

S4 Fig The average experimental measurements taken at sensor location I0. Grey bands indicate ± one standard deviation.
(TIF)

S5 Fig A comparison between different methods of simulating the incident pressures. Simulations using the cylindrical sensing apparatus to simulate the incident pressure measurements exhibited a larger underpressure.
(TIF)

S6 Fig Pressure metrics for the low strength and high strength shocks. (TIF)

S1 Table Literature survey of recent studies conducted with shock tubes. (DOCX)

S2 Table Significant differences in pressure readings. The significant differences between the peak overpressure, signal duration, and signal impulses between vertical measurements. Data were analyzed using a two-way ANOVA with Bonferroni’s multiple comparisons test. Significance was set to be 0.05.
(DOCX)
