# Supplementary figures and images for: The evolution of secondary flow phenomena and their effect on primary shock conditions in shock tubes: Experimentation and numerical model
Source: PLoS One. 2020 Jan 16;15(1):e0227125. doi: 10.1371/journal.pone.0227125 (PMC6964877; doi:10.1371/journal.pone.0227125)

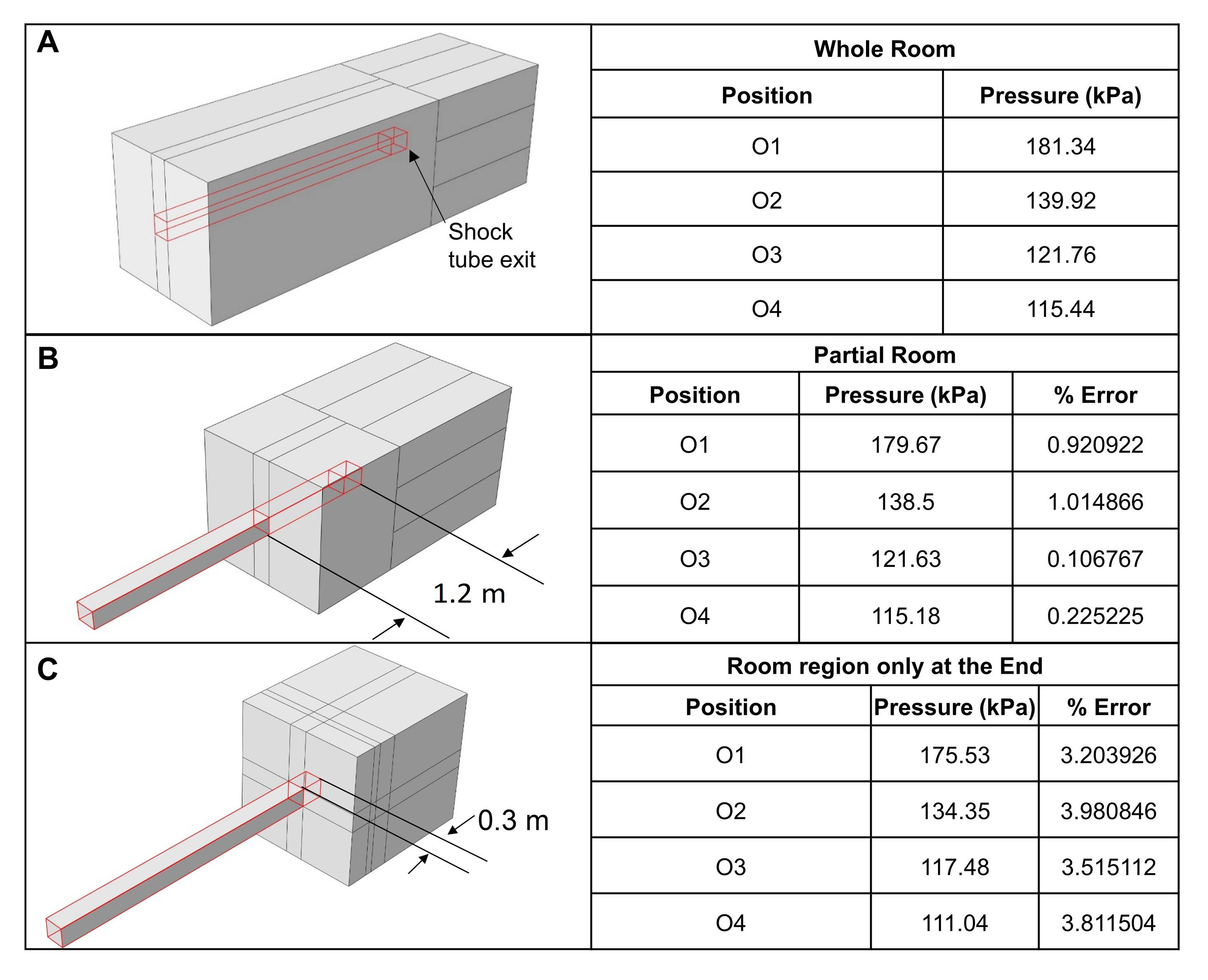

Supplement: S1 Fig — Various sizes of the room region the shock tube were modeled. The pressures at the experimental measurement locations O1-O4 were compared. Three room configurations were modeled, (A) the whole room region, (B) a partial section of the room, and (C) a room region only around the exit of the shock tube. The partial room model was selected to minimize computational errors and simulation time. (TIF) [file pone.0227125.s001.tif]

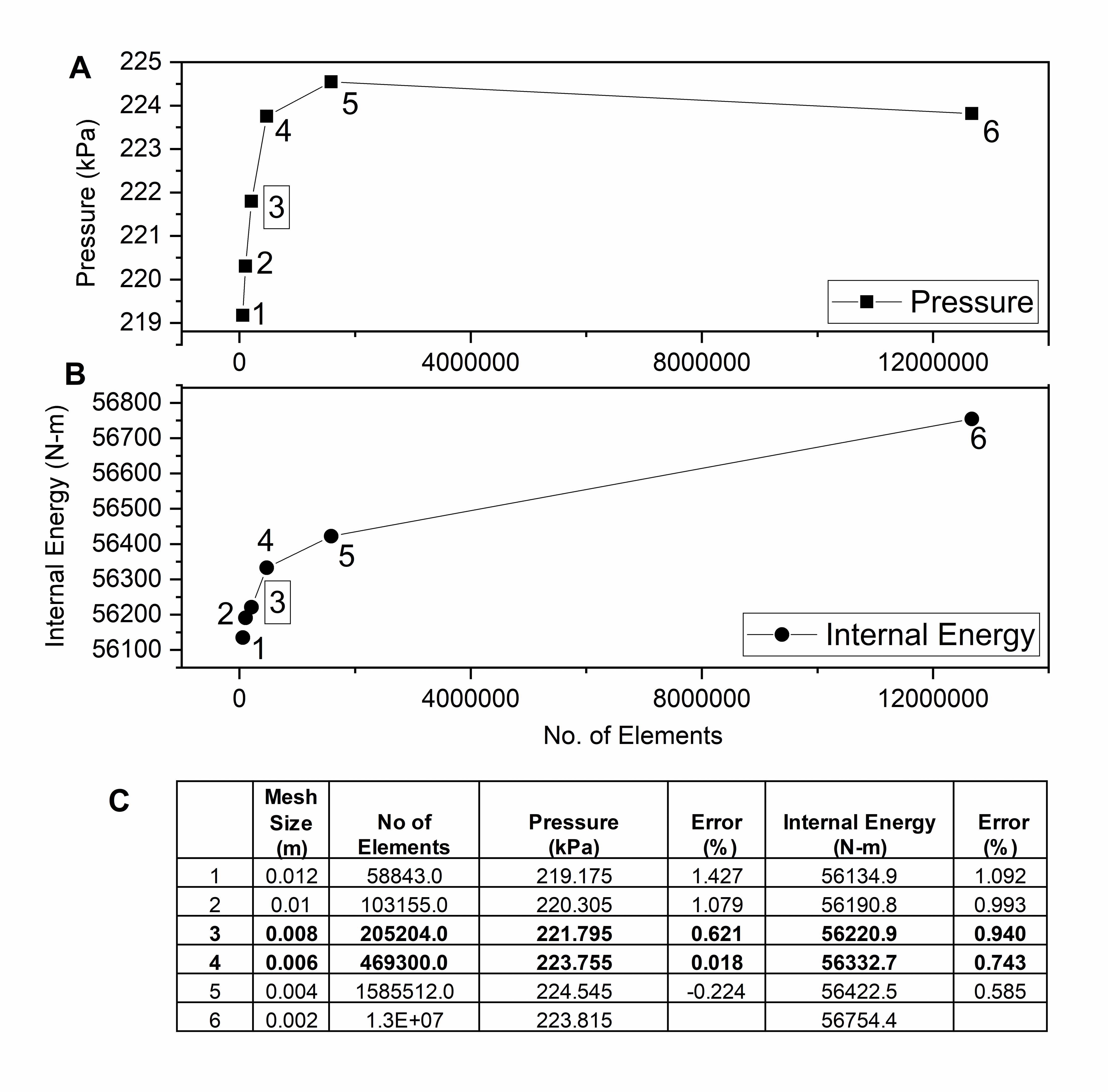

Supplement: S2 Fig — (A) The pressure at the testing location within the shock tube was plotted against the number of simulated elements. (B) The internal energy in the shock tube with respect to the number of simulated elements. (C) Table comparing the percent change between mesh seed lengths. Convergence was observed by a mesh seed length of 8 mm. (TIF) [file pone.0227125.s002.tif]

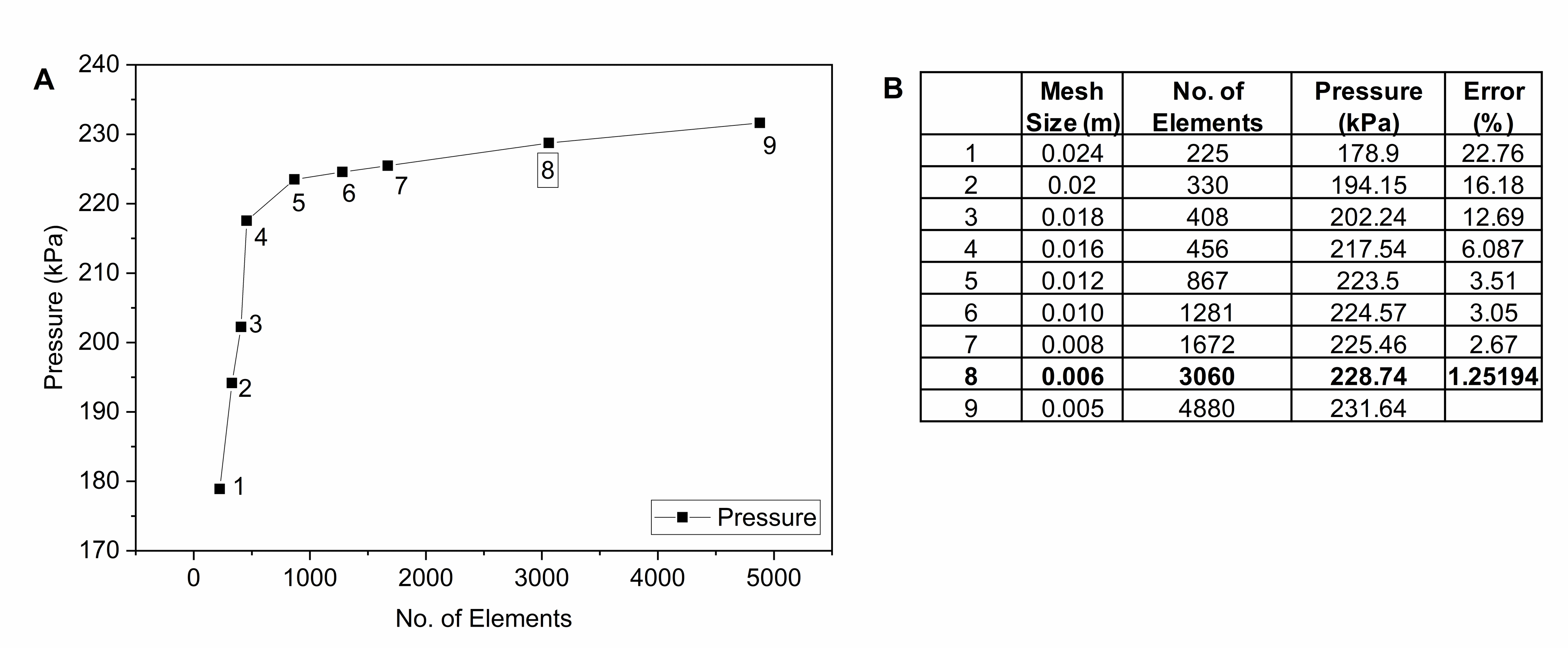

Supplement: S3 Fig — (A) The pressure at the midline of the sensing apparatus, corresponding with the longitudinal axis of the shock tube, was plotted against the number of Lagrangian elements. (B) The percent change between the pressure prediction for varying mesh seed lengths shows convergence at 6 mm. (TIF) [file pone.0227125.s003.tif]

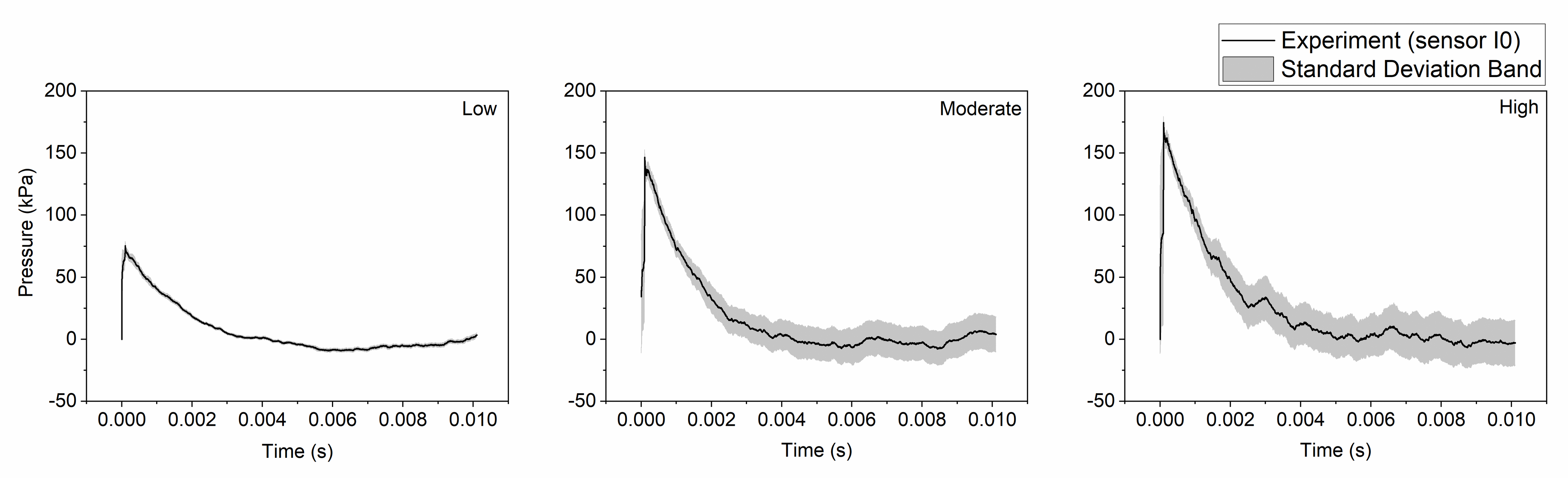

Supplement: S4 Fig — Grey bands indicate ± one standard deviation. (TIF) [file pone.0227125.s004.tif]

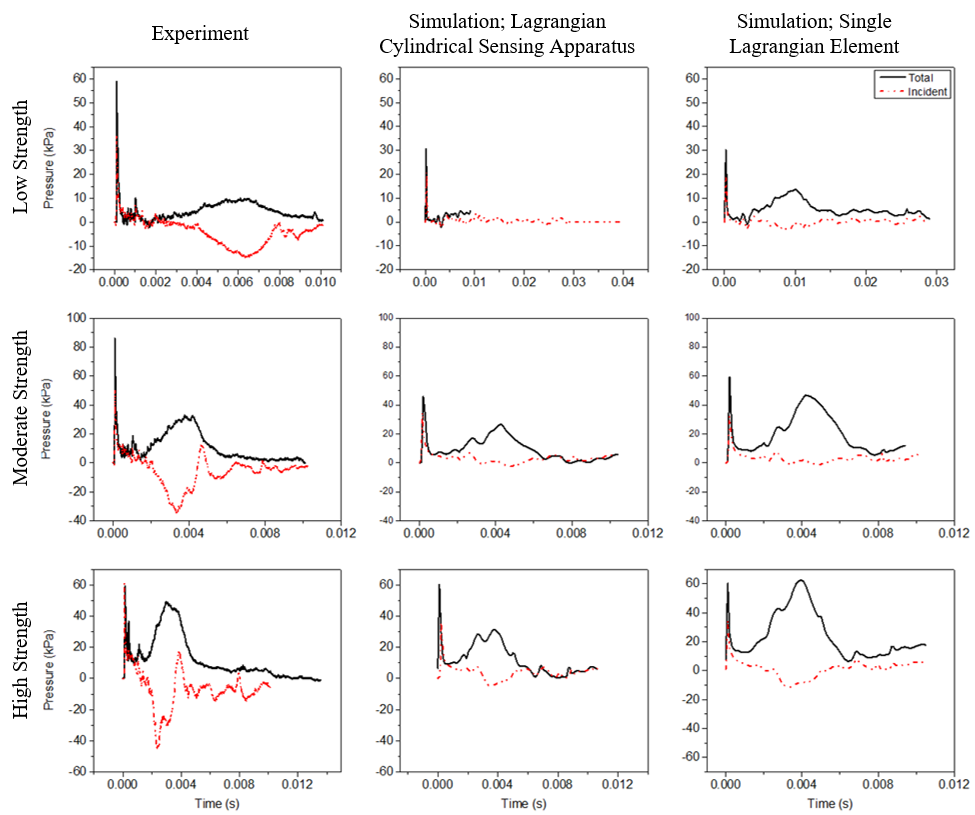

Supplement: S5 Fig — Simulations using the cylindrical sensing apparatus to simulate the incident pressure measurements exhibited a larger underpressure. (TIF) [file pone.0227125.s005.tif]

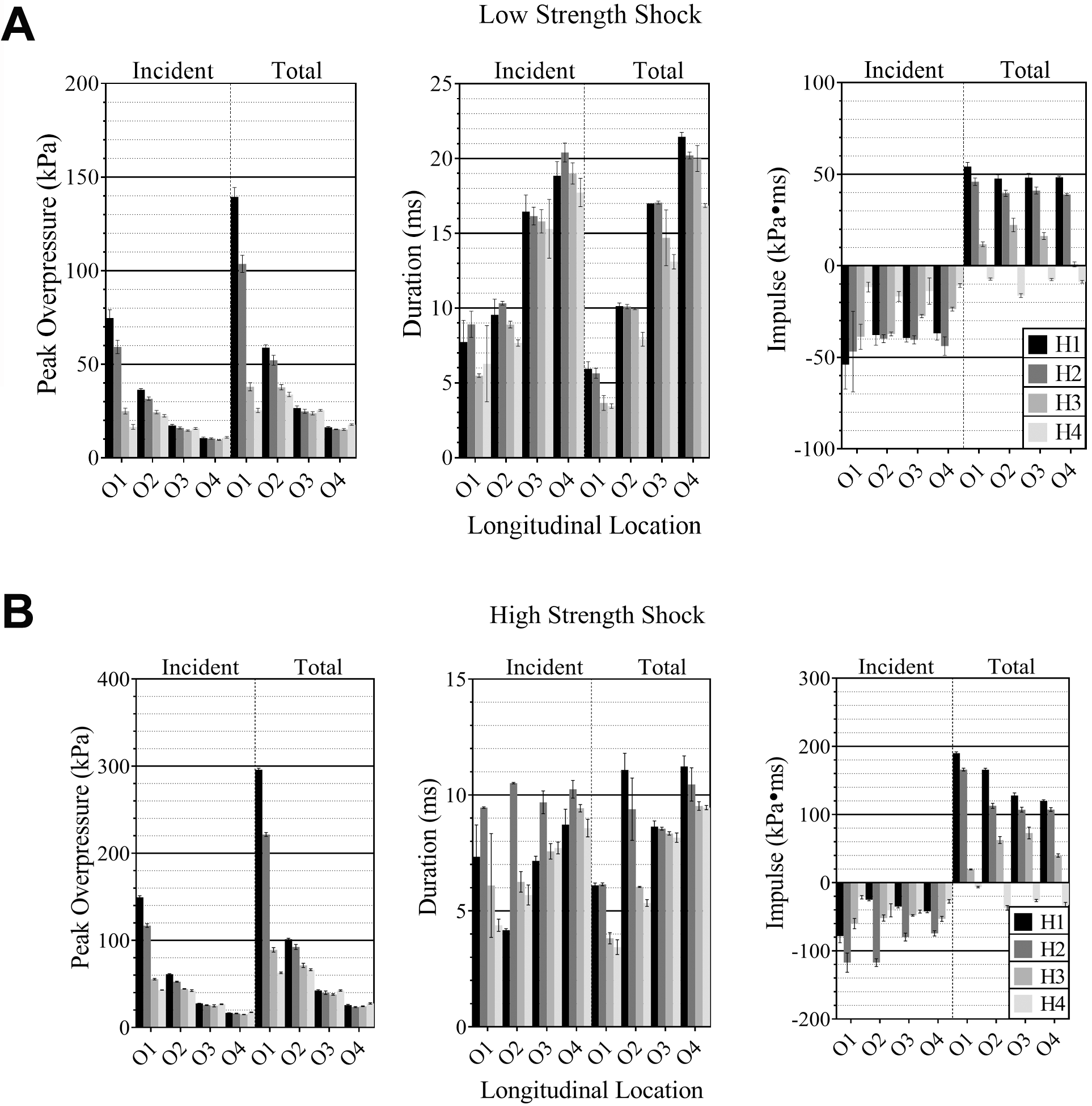

Supplement: S6 Fig — (TIF) [file pone.0227125.s006.tif]
