# Supplementary material for: The evolution of secondary flow phenomena and their effect on primary shock conditions in shock tubes: Experimentation and numerical model
Source: PLoS One. 2020 Jan 16;15(1):e0227125. doi: 10.1371/journal.pone.0227125 (PMC6964877; doi:10.1371/journal.pone.0227125)
Supplement: S1 Table — (DOCX) [file pone.0227125.s007.docx]

**S1 Table.** **Recent literature survey of studies conducted with shock tubes.**

| Study | Subject | Specimen | Shock Tube Type | Shock Strength | Specimen Location | | | Shock Tube | | | Specimen Orientation |
| --- | --- | --- | --- | --- | --- | --- | --- | --- | --- | --- | --- |
|  |  |  |  |  | IN. | END | OUT. | LENGTH | SHAPE | CSA |  |
| Courtney 2010 (1) | Shock Physics | NA | Rifle Primers | BOP: 150-700 kPa |  |  | x | NR | C | NR | NA |
| Readnower 2010 (2) | Biology | SD | CG | BOP: 120 kPa |  | x |  | 4.6 m | C | 731 cm^2^ | Lateral |
| Saljo 2010 (3) | Behavioral | Wistar | CG | BOP: 10-60 kPa | x |  |  | 3.1 m | C | 314 cm^2^ | Long. |
| Svetlov 2010 (4) | Biology | Rat | CG | BOP: 172 kPa |  |  | x | NR | C | 5.07 cm^2^ | Lateral |
| Arun 2011 (5) | Biology | Cell Cultures | CG | BOP: 94.3-145 kPa | x |  |  | 4.6 m | C | 731 cm^2^ | Lateral and Long. |
| Cernak 2011 (6) | Biology | C57/BL6 | CG | BOP: 69-105 kPa | x |  |  | 5.9 m | C | 167 cm^2^ | Long. |
| Chavko 2011 (7) | Biomechanics | SD | CG | BOP: 36 kPa | x |  |  | 5.25 m | C | 707 cm^2^ | Lateral and Long. |
| Garman 2011 (8) | Biology | SD | CG | BOP: 241 kPa |  |  | x | 6.4 m | C | NR | Lateral |
| Gyorgy 2011 (9) | Biology | Porcine | CG | BOP: 165-358 kPa | x |  |  | 21.3 m | C | 2550 cm^2^ | Long. |
| Koliatsos 2011 (10) | Biology/ Behavioral | C57/BL6 | CG | BOP: 172-310 kPa | x |  |  | 5.9 m | C | 167 cm^2^ | Vertical |
| Kuehn 2011 (11) | Biology | LE | COBIA | BOP: 260-1375 kPa |  |  | x | 9.5-29.5 cm | C | 3.30-5.07 cm^2^ | Long. |
| Leonardi 2011 (12) | Biomechanics | SD | CG | BOP: 70 kPa | x |  |  | 6.1 m | C | 731 cm^2^ | Long. |
| Mediavilla Varas 2011 (13) | Biomechanics | Human Surrogate | CG | BOP: 40 kPa | x |  |  | 13.5 m | R | 1600 cm^2^ | NA |
| Rafaels 2011 (14) | Biomechanics | NZ Rabbit | CG | BOP: 169-1090 kPa |  | x |  | 1.2 m | C | 324 cm^2^ | NR |
| Reneer 2011 (15) | Biomechanics | SD | CG | BOP: 100-200 kPa | x |  |  | 5.8 m | C | 731 cm^2^ | Lateral |
| Risling 2011 (16) | Biology | SD | Explosives | BOP: 130-260 kPa | x |  |  | 1.5 m | C | 1260 cm^2^ | Lateral |
| Ahlers 2012 (17) | Biology | LE | CG | BOP: 36.6-117 kPa | x |  |  | 5.2 m | C | 731 cm^2^ | Lateral and Long. |
| Balakathiresan 2012 (18) | Biology | SD | CG | BOP: 120 kPa |  | x |  | 4.6 m | C | 731 cm^2^ | Lateral |
| Bir 2012 (19) | Biology | SD | CG | BOP: 90-193 kPa | x |  |  | 6.0 m | C | 731 cm^2^ | Long. |
| Courtney 2012 (20) | Shock Physics | NA | OA | BOP: 204-1190 kPa |  | x |  | 26.7-30.5 cm | C | 2.01-49.0 cm^2^ | NA |
| Chandra 2012 (21) | Shock Physics | NA | CG | BOP: 150-250 kPa | x |  |  | 6.0 m | R | 529 cm^2^ | NA |
| Dalle Lucca 2012 (22) | Biology | SD | CG | BOP: 120 kPa |  | x |  | 4.6 m | C | 731 cm^2^ | Lateral |
| Elder 2012 (23) | Biology | LE | CG | BOP: 74.5 kPa |  |  | x | 5.2 m | C | 731 cm^2^ | Long. |
| Gardener 2012 (24) | Materials | Composites | CG | BOP: 1500 kPa |  | x |  | 6.2 m | C | 45.4 cm^2^ | NA |
| Goeller 2012 (25) | Biomechanics | Human Surrogate | CG | BOP: 69-170 kPa |  |  | x | 2.4 m | C | 1640 cm^2^ | NA |
| Gupta 2012 (26) | Materials | Composites | CG | BOP: 900 kPa |  | x |  | 6.2 m | C | 45.4 cm^2^ | NA |
| Houas 2012 (27) | Shock Physics | NA | CG | M: 1.06-1.25 | NA |  |  | 0.75 m | R | 64.0 cm^2^ | NA |
| Kovesdi 2012 (28) | Biology | SD | CG | BOP: 142 kPa | x |  |  | 4.6 m | C | 731 cm^2^ | Lateral |
| Shridharani 2012 (29) | Biomechanics | Porcine | CG | BOP: 110-740 kPa |  |  | x | NR | C | 731 cm^2^ | Long. |
| Sundaramurthy 2012 (30) | Biomechanics | SD | CG | BOP: 100-225 kPa | x |  |  | 6.0 m | R | 529 cm^2^ | Long. |
| Vandevord 2012 (31) | Biomechanics/ Behavioral | SD | CG | BOP: 97-153 kPa | x |  |  | 6.1 m | C | 731 cm^2^ | Long. |
| Abdul-Muneer 2013 (32) | Biology | SD | CG | BOP: 123 kPa | x |  |  | 6.0 m | R | 529 cm^2^ | Long. |
| Abotula 2013 (33) | Materials | Composites | CG | BOP: 250 kPa |  | x |  | 6.2 m | C | 45.4 cm^2^ | NA |
| Ahmed 2013 (34) | Biology | SD | CG | BOP: 138 kPa |  |  | x | 4.6 m | C | 731 cm^2^ | Lateral |
| Arun 2013 (35) | Biology | C57BL/6 | CG | BOP: 142 kPa | x |  |  | 4.6 m | C | 731 cm^2^ | Long. |
| Cho 2013 (36) | Biology | SD | CG | BOP: 129 kPa | x |  |  | 2.0 m | R | 930 cm^2^ | Long. |
| Genovese 2013 (37) | Biology | SD | CG | BOP: 74.5 kPa | x |  |  | 4.6 m | C | 731 cm^2^ | Long. |
| Huber 2013 (38) | Biology | C57/BL6 | CG | BOP: 109 kPa | x |  |  | 5.0 m | C | 167 cm^2^ | Vertical |
| Prima 2013 (39) | Biology | Rat | CG | BOP: 230-380 kPa |  |  | x | NR | C | 5.07 cm^2^ | Lateral |
| Selvan 2013 (40) | Biomechanics | Cylinder | CG | BOP: 150 kPa | x |  |  | 6 m | R | 529 cm^2^ | NA |
| Skotak 2013 (41) | Biomechanics | SD | CG | BOP: 130-290 kPa | x |  |  | 6 m | R | 529 cm^2^ | Long. |
| Turner 2013 (42) | Biomechanics | Rat | CG | BOP: 214-621 kPa |  |  | x | NR | C | 227 cm^2^ | Lateral |
| Tümer 2013 (43) | Biology | SD | CG | BOP: 358 kPa |  |  | x | NR | C | 5.07 cm^2^ | Lateral |
| Valiyaveettil 2013 (44) | Biology | C57BL/6 | CG | BOP: 142 kPa | x |  |  | 4.6 m | C | 731 cm^2^ | Long. |
| Yeoh 2013 (45) | Biology | SD | Rifle Primers | BOP: 145-323 kPa |  |  | x | 0.56 m | C | NR | Long. with offset |
| Zhu 2013 (46) | Biomechanics | Porcine | Explosive | BOP: 100-250 kPa | x |  |  | 18.3 m | C | 2630 cm^2^ | Lateral |
| Courtney 2014 (47) | Shock Physics | NA | OA | BOP: 1.7-5.3 MPa |  |  | x | 3.05 m | C | 22.1 cm^2^ | NA |
| Effgen 2014 (48) | Biology | SD Hippocampal Cultures | CG | BOP: 92.7-534 kPa |  | x |  | 1.24 m | C | 45.4 cm^2^ | NA |
| Gullotti 2014 (49) | Biomechanics | C57/BL6 | CG | BOP: 120-500 kPa |  |  | x | 1.31 m | C | 47.8 cm^2^ | Angled, Lateral, and Long. |
| Hua 2014 (50) | Biomechanics | Human Surrogate | CG | BOP: 130 kPa | x |  |  | 12.3 m | R | 5040 cm^2^ | Long. |
| Kamnaksh 2014 (51) | Biology | SD | CG | BOP: 137 kPa | NR |  |  | 4.6 m | C | 731 cm^2^ | Lateral |
| Nguyen 2014 (52) | Shock Physics | NA | CG | BOP: 200 kPa | NA |  |  | 3.8 m | C | 27.3 cm^2^ | NA |
| Simard 2014 (53) | Biology | LE | TOBIA | BOP: 462-517 kPa |  | x |  | 24.5-27 mm | C | 5.07 cm^2^ | Lateral |
| Sundaramurthy 2014 (54) | Shock Physics | NA | CG | BOP: 70-350 kPa | x |  |  | 6.8 m | R | 5040 cm^2^ | NA |
| Yazici 2014 (55) | Shock Physics | Corrugated steel | CG | BOP: 1100 kPa |  | x |  | 6.2 m | C | 11.3 cm^2^ | NA |
| Kabu 2015 (56) | Biology | SD | OA | BOP: 315-896 kPa |  |  | x | 1.8 m | C | 5.07 cm^2^ | Long. |
| Aune 2016 (57) | Shock Physics | NA | CG | M: 1.27-2.07 |  | x |  | 16.2 m | R | 900 cm^2^ | NA |
| Chan 2016 (58) | Shock Physics | Flame | CG | M: 1.10-1.48 |  | x |  | 2.2 m | R | 25.1 cm^2^ | NA |
| DeMar 2016 (59) | Biology | SD | CG | BOP: 138 kPa | x |  |  | 4.6 m | C | 731 cm^2^ | Lateral and Long. |
| Harington 2016 (60) | Biomechanics | Human Surrogate | CG | BOP: 44.8-60.7 kPa |  | x |  | 4 m | C | 2490 cm^2^ | Long. |
| Huber 2016 (61) | Biology | C57BL/6 | Explosives | BOP: 105.5 kPa | x |  |  | 5.2 m | R | NR | Vertical |
| Mishra 2016 (62) | Biology | SD | CG | BOP: 60-420 kPa | x |  |  | 6 m | R | 529 cm^2^ | Long. |
| Stemper 2016 (63) | Behavioral | SD | CG | BOP: 450 kPa |  |  | x | 3 m | C | 10.2 cm^2^ | Lateral |
| Stolz 2016 (64) | Shock Physics | NA | CG | Variable |  |  | x | 22 m | R | 90000 cm^2^ | NA |
| DeMar 2016 (59) | Biology | SD | CG | BOP: 138 kPa | x |  |  | 4.6 m | C | 731 cm^2^ | Lateral and Long. |
| Sundararaj 2017 (65) | Shock Physics/ Materials | Nylon, Steel, and Wood | CG | BP: 76-227 kPa |  | x |  | 5 m | C | 50.3 cm^2^ | NA |
| Alay 2018 (66) | Biomechanics | Human/ Rodent Surrogate | CG | BOP: 130 kPa | x |  | x | 6 m | R | 529-5040 cm^2^ | Long. |
| Marty 2018 (67) | Shock Physics | NA | CG | M: 1.12-1.69 |  | x |  | 2.99 m | R | 64.0 cm^2^ | NA |
| Medhi 2018 (68) | Shock Physics | NA | CG | M: 1.33 |  | x | x | 5 m | C | 7.55 cm^2^ | NA |

NA: Not Applicable; NR: Not Reported

LE: Long Evans Rat; NZ: New Zealand; SD: Sprague Dawley Rat

COBIA: Cranium Only Blast Injury Apparatus – .22 caliber brass cartridge and a blast dissipation chamber; CG: Compressed Gas; OA: Oxy-Acetylene Mixture; TOBIA: Thorax Only Blast Injury Apparatus – .22 caliber brass cartridge and a blast dissipation chamber

BOP: Blast Overpressure; BP: Burst Pressure; M: Mach number

Inside: Within; End: +/- 50 mm; Outside: >50 mm

R: Rectangular Cross Section; C: Circular Cross Section; CSA: Cross-Sectional Area

Long. (Longitudinal): aligned with the axis of the shock tube and the coronal plane (humans) and the transverse plane (animals)

Vertical: perpendicular to the longitudinal axis of the shock tube and the transverse plane (humans) and the frontal plane (animals)

Lateral: perpendicular to the sagittal plane (human and animal)

Angled: 45° from shock tube axis, nose pointing away from the shock tube exit (animal)
