# Supplementary material for: The evolution of secondary flow phenomena and their effect on primary shock conditions in shock tubes: Experimentation and numerical model
Source: PLoS One. 2020 Jan 16;15(1):e0227125. doi: 10.1371/journal.pone.0227125 (PMC6964877; doi:10.1371/journal.pone.0227125)
Supplement: S2 Table — The significant differences between the peak overpressure, signal duration, and signal impulses between vertical measurements. Data were analyzed using a two-way ANOVA with Bonferroni’s multiple comparisons test. Significance was set to be 0.05. (DOCX) [file pone.0227125.s008.docx]

**S2 Table. Significant differences in pressure readings.** The significant differences between the peak overpressure, signal duration, and signal impulses between vertical measurements. Data were analyzed using a two-way ANOVA with Bonferroni’s multiple comparisons test. Significance was set to be 0.05.

| **Peak Overpressure** | | | | | | | | | | | | |
| --- | --- | --- | --- | --- | --- | --- | --- | --- | --- | --- | --- | --- |
| Incident Pressure | | | | | | | | | | | | |
|  | Low Strength | | | | Moderate Strength | | | | High Strength | | | |
|  | O1 | O2 | O3 | O4 | O1 | O2 | O3 | O4 | O1 | O2 | O3 | O4 |
| H1 vs. H2 | **** | ** | ns | ns | **** | ** | ns | ns | **** | **** | ns | ns |
| H1 vs. H3 | **** | **** | ns | ns | **** | **** | ns | ns | **** | **** | * | ns |
| H1 vs. H4 | **** | **** | ns | ns | **** | **** | ns | ns | **** | **** | ns | ns |
| H2 vs. H3 | **** | **** | ns | ns | **** | ** | ns | ns | **** | **** | ns | ns |
| H2 vs. H4 | **** | **** | ns | ns | **** | **** | ns | ns | **** | **** | ns | ns |
| H3 vs. H4 | **** | ns | ns | ns | **** | ns | ns | ns | **** | ns | ns | * |
| Total Pressure | | | | | | | | | | | | |
|  | Low Strength | | | | Moderate Strength | | | | High Strength | | | |
|  | O1 | O2 | O3 | O4 | O1 | O2 | O3 | O4 | O1 | O2 | O3 | O4 |
| H1 vs. H2 | **** | **** | ns | ns | **** | **** | ns | ns | **** | **** | * | ns |
| H1 vs. H3 | **** | **** | ns | ns | **** | **** | ns | ns | **** | **** | **** | ns |
| H1 vs. H4 | **** | **** | ns | ns | **** | **** | ns | ns | **** | **** | ns | ns |
| H2 vs. H3 | **** | **** | ns | ns | **** | **** | ns | ns | **** | **** | ns | ns |
| H2 vs. H4 | **** | **** | ns | ns | **** | **** | ns | ns | **** | **** | * | **** |
| H3 vs. H4 | **** | * | ns | ns | **** | ns | ns | ns | **** | **** | **** | ** |
| **Duration** | | | | | | | | | | | | |
| Incident Pressure | | | | | | | | | | | | |
|  | Low Strength | | | | Moderate Strength | | | | High Strength | | | |
|  | O1 | O2 | O3 | O4 | O1 | O2 | O3 | O4 | O1 | O2 | O3 | O4 |
| H1 vs. H2 | ns | ns | ns | ns | ns | ** | * | ns | **** | **** | **** | ** |
| H1 vs. H3 | ** | ns | ns | ns | ns | ns | ns | **** | * | **** | ns | ns |
| H1 vs. H4 | ns | * | ns | ns | ns | ns | ns | **** | **** | ** | ns | ns |
| H2 vs. H3 | **** | ns | ns | ns | ns | ns | ns | **** | **** | **** | **** | ns |
| H2 vs. H4 | *** | *** | ns | *** | ns | ns | ns | **** | **** | **** | **** | ** |
| H3 vs. H4 | ns | ns | ns | ns | ns | ns | ns | **** | *** | ns | ns | ns |
| Total Pressure | | | | | | | | | | | | |
|  | Low Strength | | | | Moderate Strength | | | | High Strength | | | |
|  | O1 | O2 | O3 | O4 | O1 | O2 | O3 | O4 | O1 | O2 | O3 | O4 |
| H1 vs. H2 | ns | ns | ns | ns | ns | **** | ns | **** | ns | *** | ns | ns |
| H1 vs. H3 | ** | ns | ** | ns | **** | **** | ns | **** | **** | **** | ns | *** |
| H1 vs. H4 | *** | ** | **** | **** | **** | **** | **** | **** | **** | **** | ns | *** |
| H2 vs. H3 | * | ns | ** | ns | **** | ns | ns | **** | **** | **** | ns | ns |
| H2 vs. H4 | ** | ** | **** | **** | **** | ns | **** | **** | **** | **** | ns | ns |
| H3 vs. H4 | ns | ** | ns | **** | ns | ns | ns | *** | ns | ns | ns | ns |
| **Impulse** | | | | | | | | | | | | |
| Incident Pressure | | | | | | | | | | | | |
|  | Low Strength | | | | Moderate Strength | | | | High Strength | | | |
|  | O1 | O2 | O3 | O4 | O1 | O2 | O3 | O4 | O1 | O2 | O3 | O4 |
| H1 vs. H2 | ns | ns | ns | ns | **** | **** | ** | *** | **** | **** | **** | **** |
| H1 vs. H3 | *** | ns | * | ** | **** | ns | * | **** | **** | **** | ** | * |
| H1 vs. H4 | **** | **** | **** | **** | **** | * | **** | **** | **** | *** | ns | *** |
| H2 vs. H3 | ns | ns | ** | **** | **** | **** | **** | **** | **** | **** | **** | **** |
| H2 vs. H4 | **** | **** | **** | **** | **** | **** | **** | **** | **** | **** | **** | **** |
| H3 vs. H4 | **** | **** | ** | ** | **** | * | **** | ** | **** | * | ns | **** |
| Total Pressure | | | | | | | | | | | | |
|  | Low Strength | | | | Moderate Strength | | | | High Strength | | | |
|  | O1 | O2 | O3 | O4 | O1 | O2 | O3 | O4 | O1 | O2 | O3 | O4 |
| H1 vs. H2 | ns | ns | ns | ns | *** | **** | ns | ** | **** | **** | **** | ** |
| H1 vs. H3 | **** | **** | **** | **** | **** | **** | **** | **** | **** | **** | **** | **** |
| H1 vs. H4 | **** | **** | **** | **** | **** | **** | **** | **** | **** | **** | **** | **** |
| H2 vs. H3 | **** | **** | **** | **** | **** | **** | **** | **** | **** | **** | **** | **** |
| H2 vs. H4 | **** | **** | **** | **** | **** | **** | **** | **** | **** | **** | **** | **** |
| H3 vs. H4 | **** | **** | **** | ns | **** | **** | **** | **** | **** | **** | **** | **** |
